# Distribution, habitat associations, and conservation status updates for the pilose crayfish *Pacifastacus gambelii* (Girard, 1852) and Snake River pilose crayfish *Pacifastacus connectens* (Faxon, 1914) of the western United States

**DOI:** 10.7717/peerj.5668

**Published:** 2018-09-27

**Authors:** Rachel M. Egly, Eric R. Larson

**Affiliations:** Department of Natural Resources and Environmental Sciences, University of Illinois at Urbana-Champaign, Urbana, IL, United States of America

**Keywords:** Ecological niche model, *Faxonius virilis*, Signal crayfish, Boosted regression trees, Virile crayfish, Species distribution modeling, Invasive species, Exotic species, *Pacifastacus leniusculus*

## Abstract

Our study evaluates the distribution, habitat associations, and current conservation status of the Snake River pilose crayfish *Pacifastacus connectens* (Faxon, 1914) and pilose crayfish *Pacifastacus gambelii* (Girard, 1852)*,* two little-studied and data-deficient species endemic to the western United States. We first developed a species distribution model (SDM) for the pilose crayfishes based on their historical occurrence records using boosted regression trees and freshwater GIS data layers. We then sampled 163 sites in the summers of 2016 and 2017 within the distribution of these crayfishes, including 50 where these species were observed historically. We next compared our field results to modeled predictions of suitable habitat from the SDM. Our SDM predicted 73 sites (45%) we sampled as suitable for the pilose crayfishes, with a moderate AUC value of 0.824. The pilose crayfishes were generally predicted to occur in larger streams and rivers with less extreme upstream temperature and precipitation seasonality. We found the pilose crayfishes at only 20 (12%) of the 163 total sites we sampled, 14 (20%) of the 73 sites predicted as suitable for them by our SDM, and 12 (24%) of 50 historical sites that we sampled. We found the invasive virile crayfish *Faxonius virilis* (Hagen, 1870) at 22 sites total and 12 (24%) historical sites for the pilose crayfishes, and we found the “native invader” signal crayfish *Pacifastacus leniusculus* (Dana, 1852) at 29 sites total and 6 (12%) historical sites for the pilose crayfishes. We subsequently used a single classification tree to identify factors associated with our high rate of false positives for contemporary pilose crayfish distributions relative to our SDM. This classification tree identified the presence of invasive crayfishes, impairment of the benthic community, and sampling method as some of the factors differentiating false positives relative to true positives for the pilose crayfishes. Our study identified the historical distribution and habitat associations for *P. connectens* and *P. gambelii* using an SDM and contrasted this prediction to results of contemporary field sampling. We found that the pilose crayfishes have seemingly experienced substantial range declines, attributable to apparent displacement by invasive crayfishes and impairment or change to stream communities and habitat. We recommend increased conservation and management attention to *P. connectens* and *P. gambelii* in response to these findings.

## Background

North America is home to the majority of the world’s crayfish diversity, with 414 described species (Richman et al., 2015; Crandall & De Grave, 2017). However, many of these North American crayfishes are highly imperiled and at risk of extinction. Taylor et al. (2007) estimated that 48% of North American crayfishes were at some level of extinction risk, whereas a more recent International Union for the Conservation of Nature (IUCN) assessment placed 32% of North American crayfishes at risk of extinction (Richman et al., 2015). The western United States (US) is more species poor for freshwater crayfishes than the southeastern US, but its endemic genus *Pacifastacus* is representative of the conservation and management challenge for crayfishes globally. Of the *Pacifastacus* crayfishes, one is a globally cosmopolitan invasive species (the signal crayfish *Pacifastacus leniusculus*; Dana, 1852), one species is believed extinct (the sooty crayfish *Pacifastacus nigrescens*; Stimpson, 1857), another is listed as Endangered under the US Endangered Species Act (the Shasta crayfish *Pacifastacus fortis*; Faxon, 1914), and two other species, the Snake River pilose crayfish *Pacifastacus connectens* (Faxon, 1914) and the pilose crayfish *Pacifastacus gambelii* (Girard, 1852), are effectively unstudied (Larson & Williams, 2015). Currently, *P. connectens* is listed in the IUCN Red List database as Data Deficient and *P. gambelii* is listed as Least Concern (Richman et al., 2015), but no distributional or conservation status studies have been conducted for either species (Larson & Olden, 2011; Larson & Williams, 2015). Given that two species of their genus have gone extinct or been listed as Endangered, we sought to evaluate the distribution, habitat associations, and conservation status of the pilose crayfishes *P. connectens* and *P. gambelii*.

**Figure 1:**
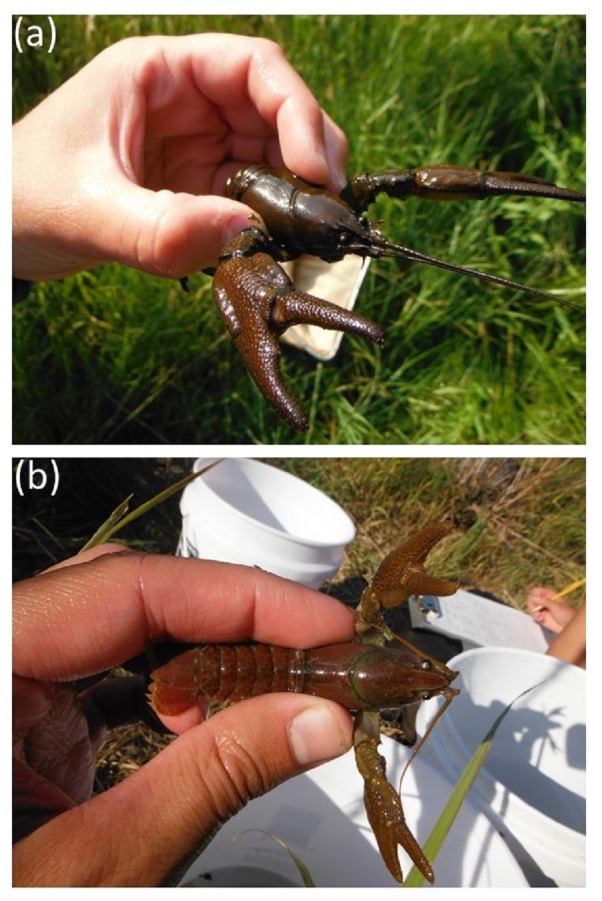
Images of *Pacifastacus connectens* (A) and *Pacifastacus gambelii* (B). These images demonstrate the acute rostrum of *P. connectens* relative to the broad rostrum of *P. gambelii*. Photos courtesy of Eric R. Larson.

*Pacifastacus connectens* and *P. gambelii* belong to the subgenus *Hobbsastacus*, which includes the extinct *P. nigrescens* and *P. fortis*, relative to the subgenus *Pacifastacus*, which includes only *P. leniusculus* and its three recognized subspecies (Larson & Williams, 2015). *P. connectens* was split from *P. gambelii*, first as a subspecies by Faxon (1914) and subsequently as its own species by Hobbs (1972). Both crayfishes are morphologically unique relative to other members of their genus owing to the presence of patches of setae or hairs on their chelae, whereas *P. connectens* is differentiated from *P. gambelii* by characteristics including an acute (narrow) rather than obtuse (broad) rostrum (Fig. 1). Recent phylogenetic species delimitation analysis has identified some ambiguity within the *Hobbsastacus* subgenus (Larson et al., 2016); as work on their taxonomic relationship continues, we largely consider both species here combined as the “pilose crayfishes” given their shared taxonomic history and morphological similarity. To date, no studies have investigated the life history or ecology of either pilose crayfish species, although Koslucher & Minshall (1973) included *P. gambelii* in a study on stream food webs from southern Idaho. Further, historical records for the pilose crayfishes appear to indicate a habitat preference for groundwater-dominated springs with small upstream catchments (Miller, 1960; Hubert, 2010).

**Figure 2:**
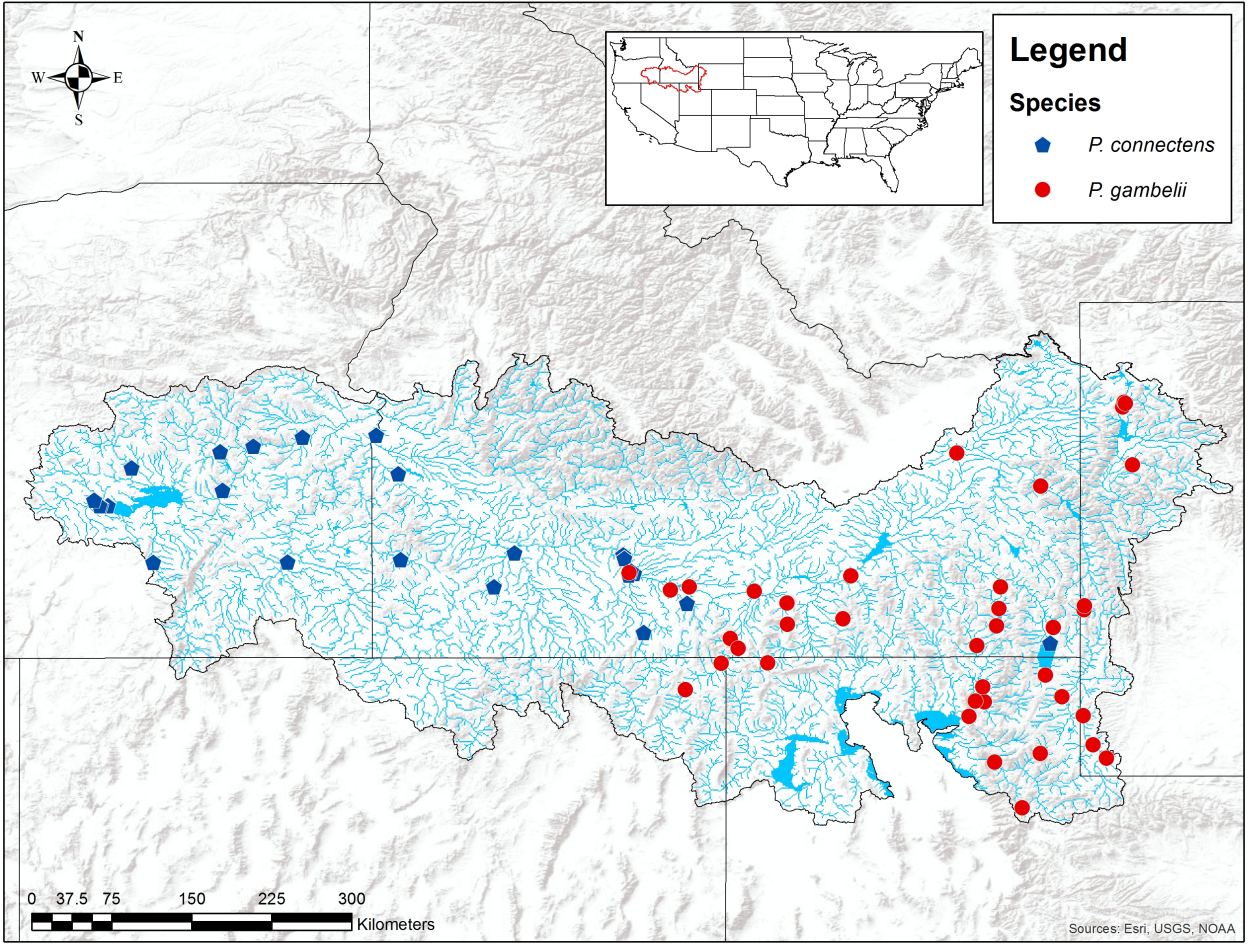
Historical *P. connectens* and *P. gambelii* occurrence records (*N* = 63). These historical occurrence records were used in species distribution modeling to identify suitable crayfish habitat (Table S1).

Data regarding the distributions of *P. connectens* and *P. gambelii* are also limited. Larson & Olden (2011) proposed the pilose crayfishes as endemic to the middle and upper Snake River drainage and adjacent closed or endorheic desert basins (e.g., the Bonneville Basin) of Idaho, Nevada, Oregon, Utah, and Wyoming (Fig. 2). Past guides or keys to North American crayfishes (e.g., Hobbs, 1972) likely over-stated the distribution of these two crayfishes, particularly *P. gambelii*, per the review of Larson & Williams (2015), although more widespread distributional surveys for these crayfishes throughout western North America would be useful. Within the range proposed by Larson & Olden (2011) and Larson & Williams (2015) for each crayfish, *P. connectens* generally occurs below Shoshone Falls, a major biogeographic break in the Snake River drainage, and in the neighboring Harney Basin of eastern Oregon. Alternatively, *P. gambelii* occurs above Shoshone Falls in the Snake River and its tributaries, and in the northern Bonneville Basin, although exceptions in this distributional pattern between the two species have been reported from historical records (Fig. 2). These erratic distributional records for each species may reflect either misidentifications in historical records or a more complex distributional pattern for each species than proposed by past work like Larson & Williams (2015), and further supports our decision to consider the two species combined here rather than separately.

Like many freshwater crayfishes, *P. connectens* and *P. gambelii* could be impacted by a number of threats and stressors within their native range (Richman et al., 2015; Bland, 2017). These include risk of displacement by invasive crayfishes, including the virile crayfish *Faxonius virilis* (Hagen, 1870) and the red swamp crayfish *Procambarus clarkii* (Girard, 1852), which have been reported as introduced in the native range of the pilose crayfishes (Johnson, 1986; Hubert, 1988; Clark & Lester, 2005); reviewed in Larson & Olden (2011). Further, the congeneric crayfish *P. leniusculus* was not known from the native range of *P. connectens* or *P. gambelii* during the earliest historical records for these species (e.g., Miller, 1960), but could represent a “native invader” (e.g., Carey et al., 2012) as it has seemingly spread inland into this region over recent decades from its more coastal native range (Larson et al., 2012). Competitive displacement by *P. leniusculus* was implicated in both the extinction of *P. nigrescens* and US Endangered Species Act listing of *P. fortis*, and *P. leniusculus* could pose a similar threat to the *Hobbsastacus* pilose crayfishes (Bouchard, 1977; Light et al., 1995). Invasive populations of *P. leniusculus* have also been attributed as a cause of declines of native European crayfish of the family Astacidae (Chucholl & Schrimpf, 2016; Maguire et al., 2018). Additionally, freshwaters of the native range of the pilose crayfishes have experienced impacts due to livestock overgrazing, flow regime modification by dams and irrigation development, and water quality impairments from agricultural and urban runoff (Belsky, Matzke & Uselman, 1999; Anderson & Woosley, 2005; Caldwell et al., 2012). In particular, the Snake River Plain has been identified as a region of hydrologic impairment and poor water quality resulting from agricultural land use (Hill et al., 2016; Thornbrugh et al., 2017). Such land use changes and their effects on water quality and freshwater habitats have similarly been attributed as contributing to native crayfish declines elsewhere (Chucholl & Schrimpf, 2016; Chucholl, 2017).

We sought to model the historical distribution and habitat associations of *P. connectens* and *P. gambelii* combined in the western US and compare these predictions to their current distribution from field sampling. We first developed a species distribution model (SDM) using historical occurrence data for *P. connectens* and *P. gambelii* to predict the distributions and habitat associations for these crayfishes using GIS environmental data layers (Domisch, Amatulli & Jetz, 2015). We then conducted field sampling in the presumed native range of these crayfishes to characterize their current distributions in comparison to both their historical occurrence records and predictions of suitable habitat by our SDM. Finally, where our SDM model predictions diverged from results of our field sampling, we used a single classification tree on factors like the presence of invasive crayfishes and GIS layers on possible stream habitat impairment to explore and explain these misclassifications. Cumulatively, our work should help to better define the historical distribution and habitat associations for the pilose crayfishes *P. connectens* and *P. gambelii*, as well as their current conservation status.

## Methods

**Figure 3:**
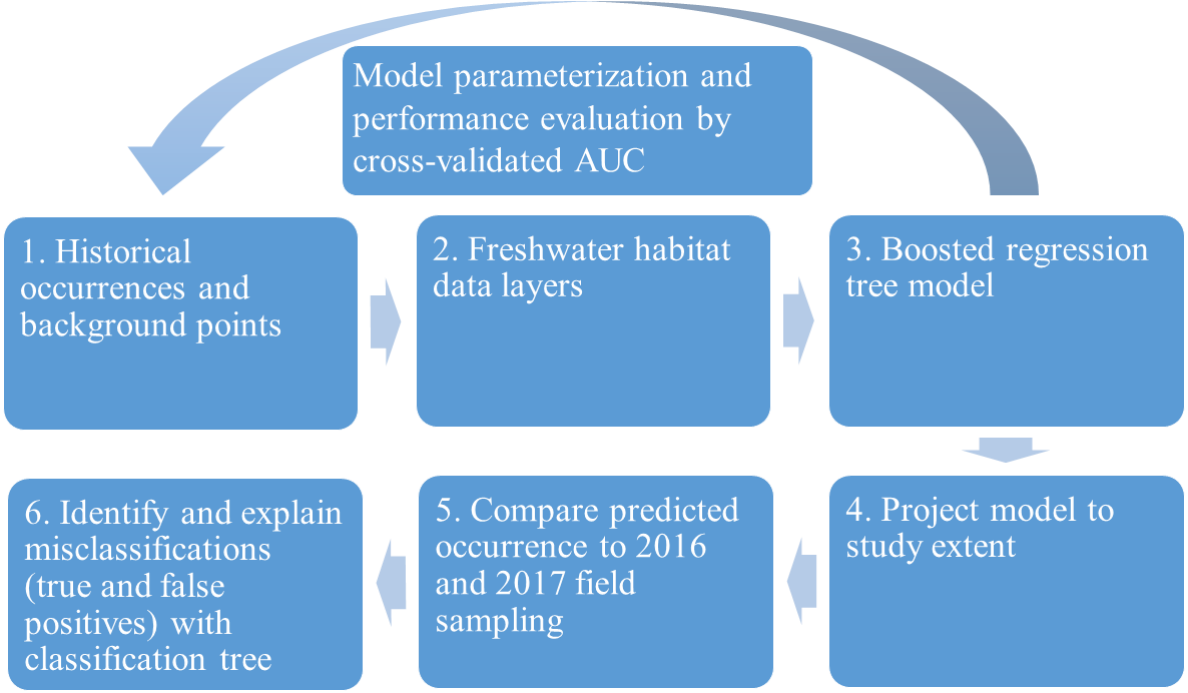
Conceptual figure representing our process. This process includes species distribution modeling (SDM), comparison to field sampling results, and explanation of classifications between the SDM and field sampling using a single classification tree differentiating true and false positives.

We evaluated the historical and current distributions and habitat associations of the pilose crayfishes *P. connectens* and *P. gambelii* using an SDM on GIS environmental data layers, along with contemporary field sampling (Fig. 3). We first used historical occurrence records for the pilose crayfishes to generate an SDM describing their past distribution and habitat associations. Upon developing this SDM, we sampled study sites predicted by our model to be suitable and unsuitable for the pilose crayfishes throughout their native range to characterize their current distribution. In relating contemporary presences or absences of *P. connectens* and *P. gambelii* to modeled predictions of habitat suitability, we anticipated that the SDM would misclassify some sampled sites. For example, false positives are places where the SDM predicted pilose crayfish to occur but we failed to find them in our field sampling. We then sought to explain such true and false positives using a subsequent, single classification tree using information like presence of invasive crayfishes at sampled sites and habitat conditions or impairment (Fig. 3).

### Species distribution modeling

We characterized the historical distribution and habitat associations for the pilose crayfishes *P. connectens* and *P. gambelii* using an SDM. We chose to combine the two pilose crayfishes in our SDM as opposed to modeling them individually due to some ambiguity in the taxonomy and geographic distributions of these two crayfishes, as well as to increase the number of historical occurrence records included in our SDM from only those for these crayfishes individually (25 for *P. connectens*; 38 for *P. gambelii*) to a greater number for both pilose crayfishes combined (63 total). Further, given the morphological and presumed ecological similarity between the two pilose crayfishes, we anticipated that a single SDM combining both species might work well, but tested performance of combined vs. separate SDMs in a series of alternative models reported in Fig. S1.

For our SDMs, we used a total of 63 historical occurrence records for *P. connectens* and *P. gambelii* identified from museum collections, government agency reports, personal communications with agency biologists, and published scientific literature, providing the best available characterization of the native ranges for these species (Fig. 2; Table S1). We also generated background (or pseudo-absence) points for the study region, which can be used to model species distributions under a number of current machine-learning methodologies when lacking true absence records (e.g., Engler, Guisan & Rechsteiner, 2004; Welk, 2004; Elith & Leathwick, 2009; Stryszowska et al., 2016). For the model reported in the main text, we used 1,000 background points generated at random within the environmental GIS layers we used constrained to the native range of these two species (see below). SDMs can be sensitive to the number and geographic distribution of background points (Engler, Guisan & Rechsteiner, 2004; Barbet-Massin et al., 2012; Mainali et al., 2015), but we found good predictive performance with this number of random background points after testing sensitivity of model results to this important decision (Fig. S1). In addition, SDMs using different combinations of background points and the two pilose crayfish species modeled separately, rather than combined, generally did not perform as well as our primary model, with significantly fewer true negatives and more false positives occurring for these models (Fig. S2).

We modeled suitable habitat for the pilose crayfishes using environmental data from the EarthEnv GIS data layers, which provide near-global freshwater-specific environmental variables in a relatively fine 1-km^2^ resolution (Domisch, Amatulli & Jetz, 2015). From these data layers, we chose environmental variables anticipated to be appropriate for historical occurrence data for *P. connectens* and *P. gambelii* (1914–2014). We chose not to include contemporary land cover data for our SDM because this variable has likely changed over recent decades, and consequently may not be appropriate for modeling distributions of historical occurrence records which date back to the early 20th century. We used as temperature variables annual mean upstream temperature (°C), upstream temperature seasonality (standard deviation of monthly average temperature in °C), maximum upstream temperature of warmest month (°C), and minimum upstream temperature of coldest month (°C). We used as precipitation variables annual upstream precipitation (mm) and upstream precipitation seasonality (coefficient of variation of monthly average precipitation in mm). We chose to include average slope (° * 100), which is averaged for each 1 km grid cell. We also included flow accumulation (count), which is the watershed area, calculated as the sum of upstream grid cells for the entire catchment delineated for each grid cell, and flow length (count), which is the length of the stream network, calculated as the sum of upstream grid cells for only the stream network within the catchment. For soil variables, we chose soil pH (pH * 10), amount of coarse fragments (% of soil above a 2 mm threshold), cation exchange capacity (cmol/kg), and depth to bedrock (cm). We anticipated that like many other crayfish species, the pilose crayfishes might have substrate preferences, particularly for coarser rock or substrate (Capelli & Magnuson, 1983; Garvey et al., 2003), and also anticipated that these crayfishes might be sensitive to the acidity or pH of water (DiStefano et al., 1991; Edwards, Jackson & Somers, 2014).

We modeled suitable habitat for the pilose crayfishes *P. connectens* and *P. gambelii* using their historical occurrence records and background points with the above environmental predictors using boosted regression trees (Elith & Leathwick, 2017). Boosted regression trees relate response variables to predictor variables using binary recursive splits and offer improved predictive ability through boosting, which creates and averages many different models (Elith, Leathwick & Hastie, 2008). Boosted regression trees characterize habitat associations and distributions for species, often from presence-only records such as those available for *P. connectens* and *P. gambelii,* and generally perform comparably to other machine-learning approaches to SDMs like MaxEnt or artificial neural networks (Elith et al., 2006).

We fit boosted regression tree models using the packages “dismo” and “gbm” in R version 3.3.2 (Ridgeway, 2015; Hijmans et al., 2017). We regularized our boosted regression tree models following the suggestions of Elith, Leathwick & Hastie (2008) in choosing learning rate, tree complexity, and bag fraction settings. Learning rate determines the contribution of each regression tree as it is added to the model, where a lower learning rate increases the number of total trees in an ensemble model; tree complexity is the number of nodes or splits in individual trees and controls the complexity of the model; and bag fraction specifies the proportion of training data to be selected at random, without replacement, for each step. For our model regularization we started with the range of values suggested by Elith, Leathwick & Hastie (2008) and narrowed down iteratively to determine the model regularization that provided the highest area under the curve (AUC) statistic for model performance by classification. AUC is the area under the curve of the receiver-operator characteristic plot, which is a measure of model classification performance for presence/absence data (Guisan & Zimmermann, 2000; Wenger & Olden, 2012; Jiménez-Valverde, 2012). AUC generally ranges from a random value of 0.5, which indicates random discrimination between presence and absence in classifying categorical variables, to a value of 1.0 which indicates all presences and absences are correctly classified at all model thresholds.

For our SDM presented in the main text (1,000 background points, with the two species combined), we ran our boosted regression tree model with a learning rate of 0.001, tree complexity of 3, and a bag fraction of 0.5. This model had a higher AUC than models for each crayfish individually, or for different numbers of background points (Fig. S1). We then projected model predictions of our best model to the full range extent of the pilose crayfishes to characterize their distributions based on historical occurrence records. We determined a threshold for habitat suitable vs. unsuitable for these crayfishes by using an optimal balance between sensitivity (true positive rate) and specificity (true negative rate) based on training data in model regularization (Elith & Leathwick, 2017). We also generated partial dependence plots for the environmental variables most important in determining crayfish occurrence from our model to characterize habitat associations for *P. connectens* and *P. gambelii*.

### Field sampling

We sampled a total of 163 sites in Idaho, Nevada, Oregon, Utah, and Wyoming anticipated to be within the native range of *P. connectens* and *P. gambelii*, with 78 sites sampled between July 16th and August 10th 2016, and 85 sites sampled between July 2nd and August 3rd 2017. We sampled 50 of the 63 historical occurrence records for the pilose crayfishes (Table S1) used in our SDM; we could not access all historical occurrences due to land ownership permissions and time constraints in some cases. Due to logistical constraints of the field sampling protocol, we opted not to randomize sampling locations, but we deliberately sought to sample a range of habitat types from small streams to large rivers and natural lakes to reservoirs.

Sites were sampled by one of two methods: either hour long timed searches by two observers (106 sites), or overnight baited trapping (57 sites). In most cases, choice of timed search or baited trapping was *ad hoc* in response to our schedule that day, although baited trapping was sometimes required at sites where timed searches were not feasible (below)*.* Timed searches used hand nets, D-frame nets, or seines depending on habitat size or other attributes. We used hand nets in the smallest streams where larger nets were difficult to use, and to search the wadeable littoral zones of reservoirs and lakes by overturning potential crayfish shelter like cobble and large woody debris. We used D-frame nets and seines in larger wadeable streams and rivers, following an approach approximating quantitative kick seining for crayfishes (Engelbert, Taylor & DiStefano, 2016). Timed searches generally covered approximately 100–200 m of linear habitat in either lotic or lentic environments. At some sites—including those too deep, too steep, or with too limited public access to sample by our timed search methods—we set crayfish traps (0.42 m long by 0.21 m diameter with two 60-mm openings) overnight that were baited with dry dog food (Larson & Olden, 2016). When trapping, we set four to six traps per site for approximately 16 h at depths ranging from a half meter to several meters deep. Field sampling was conducted under Wyoming Game and Fish scientific collecting permit 33-1070, Idaho Department of Fish and Game permits F-16-32-16 and F-16-32-17, Utah Division of Wildlife Resources permit 2COLL9870, Nevada Department of Wildlife permit 428773, and Oregon Department of Fish and Wildlife permit 21325.

### Explaining misclassifications

We anticipated that our SDM identifying suitable habitat for the pilose crayfishes might misclassify some presences and absences from our field sampling in 2016 and 2017. These misclassifications could include false negatives and false positives. In our study, false negatives are sites where the model predicted the pilose crayfishes to be absent but where we found them during field sampling, whereas false positives are sampled sites where the model predicted the pilose crayfishes to be present but we did not detect them during field sampling. False positives in particular might occur if the pilose crayfishes have experienced range and population declines in response to habitat degradation and loss or displacement by invasive crayfishes. We sought in particular to explore factors differentiating true positives, where our SDM and field sampling agreed on the presence of pilose crayfishes, from false positives using a single classification tree with predictors that could explain population or range declines for our native crayfishes, as well as potential differences in detection probability between our two sampling methods. We did not model true and false negatives, because true negatives—where habitat was not predicted to be suitable for our focal crayfishes—were not of interest for range declines, and false negatives were relatively rare and accordingly difficult to model due to low sample sizes (see ‘Results’).

We chose as predictors for this classification tree the presence of invasive crayfish (including the presumed native invader *P. leniusculus*), whether the site was a reservoir or not (measured according to waterbody classification in the National Hydrography Dataset; United States Geological Survey, 2013), sampling method, a modeled measure of stream benthic community condition (Hill et al., 2016), an estimate of stream hydrologic regulation by dams and water diversions (Hill et al., 2016), and percent of upstream urban and agricultural land cover from Domisch, Amatulli & Jetz (2015). We anticipated that presence of invasive crayfish could result in a greater number of false positives relative to SDM predictions for native pilose crayfish occurrence, since invasive crayfish commonly displace native crayfishes through mechanisms such as competition (Chucholl & Schrimpf, 2016; Maguire et al., 2018). We also expected that the pilose crayfishes might be more likely to be absent in reservoirs due to the substantial abiotic and biotic changes associated with stream and river impoundment, which may explain greater numbers of false positives in these environments (Gido, Matthews & Wolfinbarger, 2000; Johnson, Olden & Vander Zanden, 2008). We included our sampling methods as a categorical predictor for false and true positives, because we suspected that our baited trapping may have had lower detection probabilities for crayfishes in this study system than timed searches (Fig. S3), and as such choice of sampling method might explain false positives at some sites.

Stream benthic community condition is predicted by a model based on results from US Environmental Protection Agency’s (EPA) 2008/2009 National Rivers and Streams Assessment. This index is measured between 0 and 1 (where 0 is most degraded and 1 is most intact), and is predicted for each stream segment by metrics including macroinvertebrate and fish indexes, water quality, and physical habitat (Hill et al., 2016). Similarly, hydrologic regulation is an index between 0 and 1 (where 0 is highly regulated and 1 is unregulated) and is evaluated for each catchment by metrics such as upstream dam density, water use, and length and density of canals (Hill et al., 2016). We expected that poorer stream benthic community condition would result in a greater number of false positives relative to SDM predictions of pilose crayfish occurrence since it may reflect poor water quality, habitat degradation, or fewer food sources for crayfish (Momot, 1984; Bilotta & Brazier, 2008). Likewise, we expected that increased hydrological regulation would increase false positives due to possible crayfish intolerance to systems with greater alteration of the flow regime (Poff et al., 2007). We similarly expected that the pilose crayfishes might have negative relationships, and accordingly false positives relative to their historical distribution, to upstream urban and agricultural land cover, as has been observed for some other freshwater species (Allan, 2004), including crayfish (Chucholl & Schrimpf, 2016; Chucholl, 2017). We modeled our classification tree in the R package “rpart” (Therneau, Atkinson & Ripley, 2018) using a minimum split parameter of 10 and a complexity parameter of 0.01. By using this classification tree to differentiate true positives from false positives, we hoped to identify potential reasons for misclassification between our SDM based on historical occurrence data and our field sampling results.

## Results

**Figure 4:**
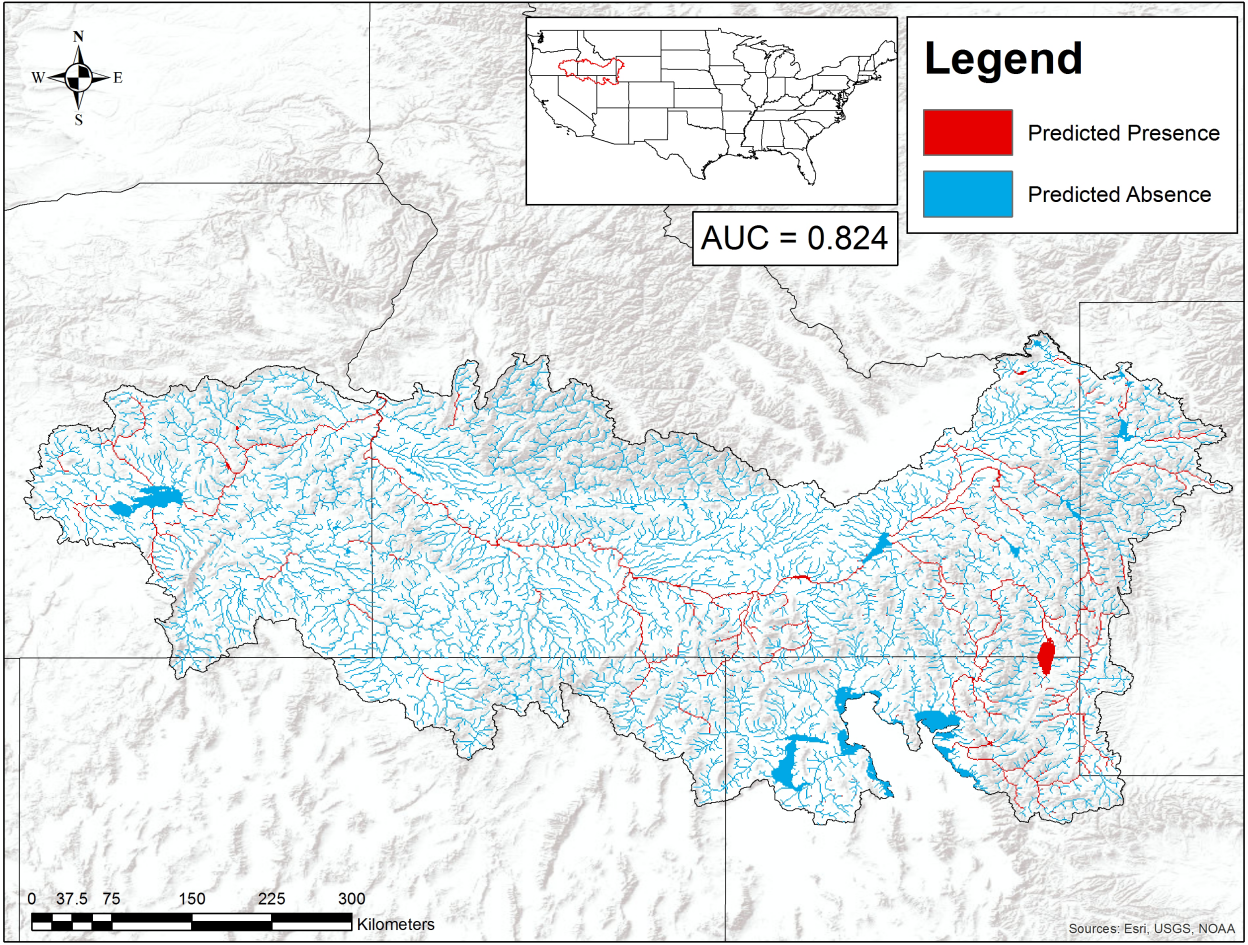
Suitable habitat for *Pacifastacus connectens* and *Pacifastacus gambelii* (combined) in the western US. Habitat was predicted from a boosted regression tree model using historical crayfish occurrence records (Fig. 2) and the EarthEnv habitat layers (Domisch, Amatulli & Jetz, 2015). The 0.107 threshold for suitable crayfish habitat is based on a balance between the true positive and true negative rate for crayfish occurrences and background points.

**Figure 5:**
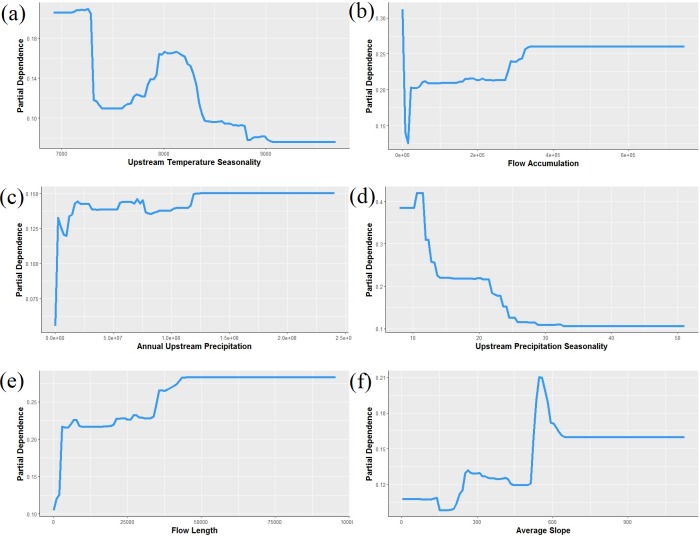
Partial dependence plots showing the relationship between the top six predictors for *Pacifastacus connectens* and *Pacifatacus gambelii* presence (combined) in the western US. (A) Upstream temperature seasonality is the standard deviation of monthly average temperature in °C. (B) Flow accumulation (count) is the watershed area, calculated as the sum of upstream grid cells for the entire catchment delineated for each grid cell. (C) Annual upstream precipitation is measured in mm. (D) Upstream precipitation seasonality is a coefficient of variation of monthly average precipitation in mm. (E) Flow length (count) is the length of the stream network, calculated as the sum of upstream grid cells for only the stream network within the catchment. (F) Average slope, averaged for each 1 km grid cell, is measured in degrees * 100.

Our boosted regression tree model classified combined *P. connectens* and *P. gambelii* historical occurrences relative to background points with a moderate AUC of 0.824 based on testing data withheld in ten-fold cross-validation (Fig. 4). Our most important environmental variables from our primary model included upstream temperature seasonality (relative importance of 13%), flow accumulation (12%), annual upstream precipitation (11.6%), upstream precipitation seasonality (11%), flow length (10%), and average slope (8%). Based on our SDM, the pilose crayfishes had a mostly negative relationship with the smallest streams in our study region, as measured by environmental variables including annual upstream precipitation, average slope, and flow length (Fig. 5). However, flow accumulation showed a positive association with some very small streams, with lower values of flow accumulation predicting a high likelihood of pilose crayfish occurrence. The pilose crayfishes also had a negative relationship with high annual upstream temperature and precipitation seasonalities.

**Figure 6:**
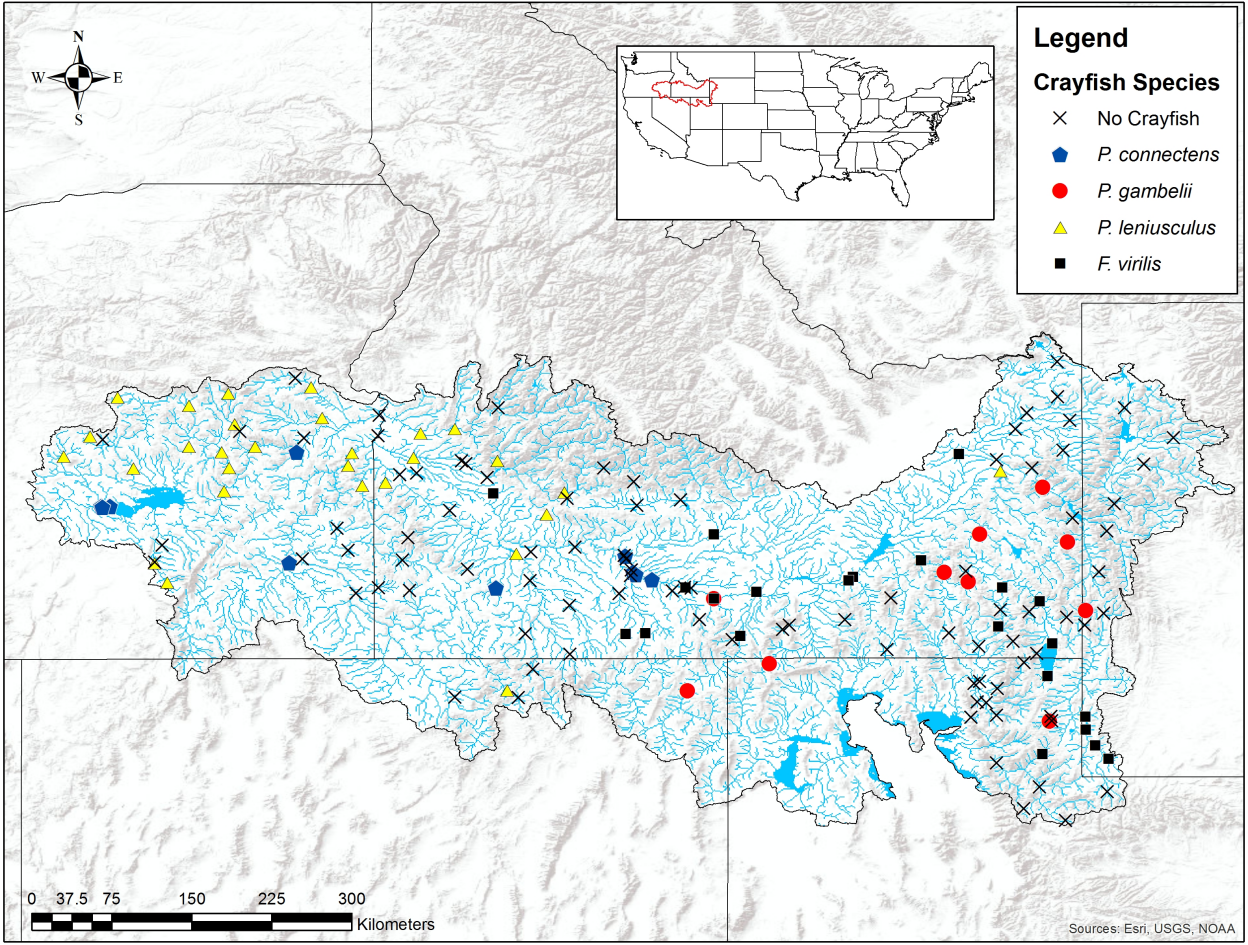
Results of field sampling for crayfish in the western US in the summers of 2016 and 2017. Crayfish species found include *Pacifastacus connectens* (*N* = 10), *Pacifastacus gambelii* (*N* = 10), *Faxonius virilis* (*N* = 22), ** and *Pacifastacus leniusculus* (*N* = 29; Table S2).

We found the pilose crayfishes at 20 (12%) of the total 163 sites we sampled, with *P. connectens* and *P. gambelii* each at 10 (Fig. 6; Table S2). We found the native invader *P. leniusculus* at 29 sites (18%) and the invasive virile crayfish *F. virilis* at 22 sites (13%), with only one site where any two crayfish species occurred sympatrically (*F. virilis* and *P. gambelii*; Fig. 6). Of the 50 historical sites we sampled, we found the pilose crayfishes at only 12 (24%), but we found *F. virilis* at 12 (24%) and *P. leniusculus* at 6 (12%). Our boosted regression tree model predicted presences and absences of pilose crayfishes from field sampling with relatively low success, based on a Cohen’s Kappa (K) of 0.14. Of the 163 sites we sampled, 73 (45%) were classified as suitable pilose crayfish habitat from our boosted regression tree model. Overall, our model correctly predicted 14 out of 20 (70%) presences for these native crayfishes (true positives), but misclassified 6 (30%) presences as unsuitable habitat for these crayfishes (false negatives). Similarly, our model correctly predicted 84 out of 143 (59%) absences (true negatives), but misclassified 59 (41%) absences as suitable habitat for *P. connectens* and *P. gambelii* (false positives).

**Figure 7:**
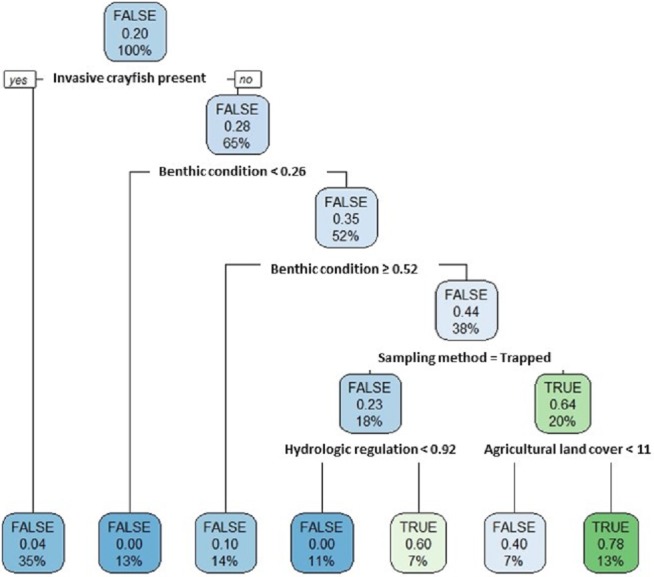
Classification tree of pilose crayfish true and false positives. This classification tree sought to differentiate false positives from true positives in comparing predictions from an SDM (Fig. 4) to contemporary field sampling (Fig. 6) for the crayfishes *P. connectens* and *P. gambelii*. Each node displays the classification (FALSE/TRUE) based on majority rule, the decision (yes/no; where yes is sorted to the left and no is sorted to the right, as demonstrated in the first node), the proportion of observations that are true positives, and the percentage of total observations (*N* = 73) present at that node. Predictor variables used at each split in the tree are given (see main text for details).

Our single classification tree differentiated false positives from true positives relatively well with a Cohen’s K of 0.64 (Fig. 7). False positives were more likely to occur at sites where invasive crayfish were present; at sites with either very poor or very good stream benthic community conditions; at sites where we used baited trapping rather than timed searches; and at sites with both greater hydrologic regulation as well as lower upstream agricultural land cover (Fig. 7).

## Discussion

We modeled suitable habitats for the pilose crayfishes *P. connectens* and *P. gambelii* based on their historical occurrence records using boosted regression trees and a series of environmental variables. We found that these crayfishes occurred historically in larger streams and rivers with lower upstream precipitation seasonality, low to intermediate upstream temperature seasonality, and higher annual upstream precipitation. We interpret these results as suggesting that the pilose crayfishes did not generally occur in high elevation, montane streams with extreme seasonality like the Uinta and Teton mountains. When related to contemporary, conventional field sampling, we found that the pilose crayfishes had seemingly experienced large population and range declines. For example, we found the pilose crayfishes at only 24% of the 50 historical occurrence records we sampled, and at only 19% of sites that our SDM predicted as suitable for them. In many cases, these declines appear attributable to displacement by the invasive crayfishes *F. virilis* and *P. leniusculus* and degraded stream benthic community condition, but choice of sampling method may also have affected the frequency of false positives we observed for the pilose crayfishes relative to modeled habitat suitability. Regardless, the pilose crayfishes seemingly require increased management and conservation attention, because they may be at risk of the types of population declines or even extinction that have been observed for similar crayfishes of the subgenus *Hobbsastacus* (Bouchard, 1977; Light et al., 1995).

**Figure 8:**
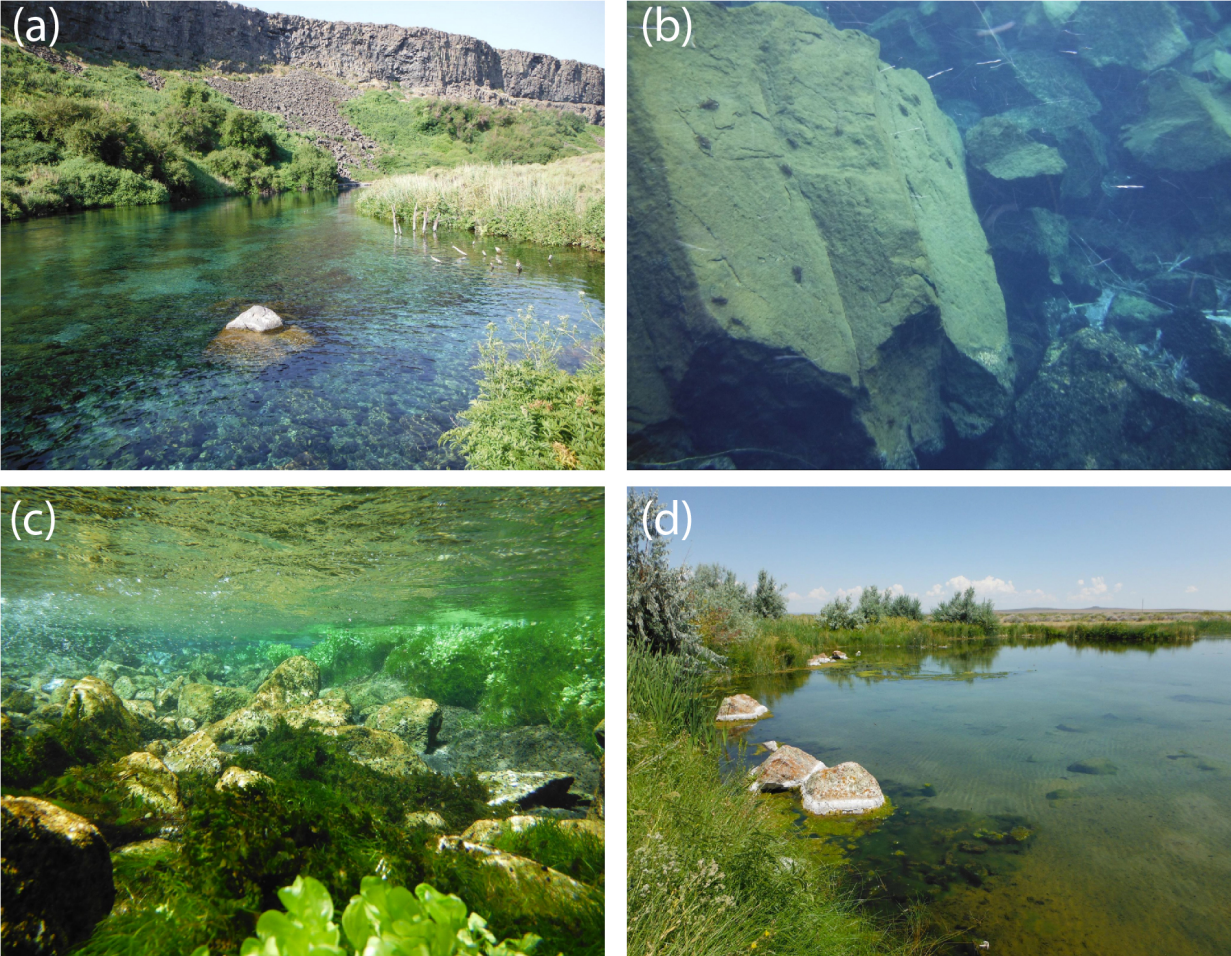
Representative habitats for *P. connectens* and *P. gambelii*. Although we found *P. connectens* and *P. gambelii* in other lentic and lotic habitat types, these crayfishes often occur in groundwater-dominated spring systems with small upstream surface watersheds, which are relatively common in the Snake River Plain. Use of these habitats explains the relationship between presence of the pilose crayfishes and streams with extremely low upstream flow accumulation (Fig. 5), as well as some false positives in comparison of our SDM (Fig. 4) to field sampling results (Fig. 6), due to the likely inaccuracy of GIS data in representing conditions for these groundwater springs. Examples include Box Canyon Spring, Idaho (A, B), Niagara Spring, Idaho (C), and springs in the vicinity of the Malheur National Wildlife Refuge, Oregon (D). Many *P. connectens* individuals are visible foraging on a boulder mid-day in (B). Photos courtesy of Eric R. Larson.

We found from our SDM that the pilose crayfishes *P. connectens* and *P. gambelii* occurred historically in larger streams and rivers in less extreme environments, featuring moderate to low temperature seasonality, low precipitation seasonality, and moderate slopes. Based on this model, the pilose crayfishes did not generally occur in the absolute smallest streams in our study region, as measured by predictors like flow accumulation, annual upstream precipitation, and flow length. Previous studies have found other crayfish species to either favor or disfavor smaller and potentially intermittent streams due to different tolerances to abiotic factors like stream drying and biotic factors like longitudinally structured predator communities (Flinders & Magoulick, 2005; Creed, 2006). However, as an exception to our finding that pilose crayfishes did not historically occur in the smallest streams, we did find some positive association between these crayfishes and the absolute smallest streams in our region as measured by flow accumulation. This likely reflects the known tendency for these crayfishes to occur in some small, groundwater-dominated springs with minimal upstream surface watersheds (Miller, 1960; Hubert, 2010). Our contemporary field sampling similarly supported an association of the pilose crayfishes with some groundwater-dominated spring habitats (Fig. 8), which parallels habitat use of the similar and endangered *P. fortis* in northern California (Light et al., 1995). These isolated spring systems should perhaps be priorities for pilose crayfish conservation, as they have represented strongholds against displacement by invasive crayfishes for *P. fortis* (Cowart et al., 2018).

The pilose crayfishes also showed a negative relationship to streams with high upstream temperature and precipitation seasonalities, which reflect those streams and rivers draining high elevation mountain ranges in our study region, where winters are extremely wet and cold relative to warm and dry summers. Such locations likely have high spring and summer stream discharge owing to snowmelt-dominated flow regimes, as well as lower stream temperatures relative to valley bottom streams (Reidy Liermann et al., 2012). Invasive *P. leniusculus* experienced declines in abundance following high flow years in the similar Sierra Nevada mountains of California (Light, 2003), and the congeneric pilose crayfishes may be similarly intolerant of higher stream flows or discharge associated with ultra-snowmelt systems. Despite this, we did find a positive association between the pilose crayfishes and moderate upstream slope, and these crayfishes may do better in slightly higher gradient streams that maintain the type of cobble rock substrate that many crayfish species prefer as habitat (Garvey et al., 2003; Nyström et al., 2006). Our SDM revealed a number of potential habitat associations for the pilose crayfishes based on historical occurrence records at a relatively coarse 1 km^2^ spatial grain, but much more work needs to be done in order to understand the habitat preferences of these crayfishes from micro-habitat (e.g., 1 m^2^) to reach scales (e.g., 100 m; Flinders & Magoulick, 2007; Wooster, Snyder & Madsen, 2012). Such finer-grain habitat work may, in part, clarify the frequency of false positives we observed for these crayfishes when comparing SDM predictions to contemporary field sampling.

Overall, our SDM on historical occurrence records for *P. connectens* and *P. gambelii* predicted contemporary distributions for these crayfishes with relatively low success in comparison to our field sampling, with many false positives but comparatively few false negatives. Because false positives may represent range declines for the pilose crayfishes, whereas false negatives were seemingly locations where GIS data simply did not reflect in-stream conditions well (e.g., groundwater springs; Fig. 8), we sought to explain false positives relative to true positives. We did this by using a single classification tree on a series of predictors either related to factors potentially causing range declines for the pilose crayfishes based on past studies in other crayfish species (Twardochleb, Olden & Larson, 2013; Richman et al., 2015), or predictors related to possible differences in detection probabilities between our field sampling methods (Larson & Olden, 2016). We found that the best explanation for false positives for the pilose crayfish was presence of an invasive crayfish species at the site. This is consistent with many past studies which have found displacement by invasive crayfishes to be a leading driver of native crayfish population declines (Lodge et al., 2000; Pintor, Sih & Bauer, 2008), and is consistent with causes of imperilment or extinction for other *Pacifastacus* crayfishes (Bouchard, 1977; Light et al., 1995), as well as native crayfishes of the family Astacidae in Europe (Chucholl & Schrimpf, 2016; Maguire et al., 2018). The second best explanation for false positives for the pilose crayfishes was highly degraded stream benthic community condition; these crayfishes have seemingly experienced range declines at locations where stream communities are the most impaired, including many lower elevation valley bottoms which have experienced high agricultural and urban development in this region (Hill et al., 2016). Water quality impairment from agricultural and urban land use has similarly been attributed as a cause for native crayfish declines in regions like Europe (Chucholl & Schrimpf, 2016; Chucholl, 2017). We also found that false positives for the pilose crayfishes were more likely to occur at locations where we sampled by baited trapping, rather than locations where we conducted timed searches. Different sampling methods can have different detection probabilities for crayfishes across habitat types (Larson & Olden, 2016), and in this case, we routinely only collected one to two crayfish with four to six baited traps effort overnight, whereas timed searches routinely collected higher numbers of crayfish over an hour of effort (Fig. S3). As such, we recommend that future studies focused on the pilose crayfishes use timed searches where possible, and if requiring the use of baited trapping, increase trap effort (number of traps) to improve detection probabilities with this method. Finally, false positives were associated with some habitat variables that we cannot necessarily explain as being associated with likely range or population declines for the pilose crayfishes. Specifically, false positives were associated with some sites of very high stream benthic community condition (unimpaired) and were also associated with sites with low agricultural land cover. Again, we propose that better understanding of micro- to reach-scale habitat associations for the pilose crayfishes might improve our understanding of some of these false positives where we failed to find these species at places predicted suitable for them (Flinders & Magoulick, 2007; Wooster, Snyder & Madsen, 2012).

Importantly, our finding of potentially large range declines for *P. connectens* and *P. gambelii* is dependent not only on comparison to modeled suitable habitat from an SDM, but also direct comparison to historical occurrence sites that we resampled. Our SDM estimated an 80% range decline for the pilose crayfishes, whereas comparison to the 50 historical sites we re-sampled found a similar 76% range decline (63% for *P. connectens* and 85% for *P. gambelii*). We found the pilose crayfishes at only 24% of the historical sites we resampled, and in another parallel to our SDM and single classification tree results, invasive crayfishes again appeared to be a major driver of this range decline. Thirty-six percent of the 50 historical sites that we resampled were instead occupied by invasive crayfishes, with only one site where a native crayfish species (*P. gambelii*) occurred in sympatry with an invasive crayfish species (*F. virilis*). Per IUCN extinction risk assessments, range declines of ≥70% over 10 years or three generations qualify for Endangered status, whereas range declines of ≥50% over the same time periods qualify for Vulnerable status (IUCN Species Survival Commission, 2012). We do not necessarily know the rate at which pilose crayfishes have experienced population declines or range retractions, but propose that neither of the pilose crayfishes are necessarily secure from some extinction risk due to impacts of invasive crayfishes or other stressors associated with habitat loss or degradation. We recommend that state, federal, and international agencies consider elevated conservation status categories for both pilose crayfishes.

Our finding of large apparent declines in the distribution of the pilose crayfishes suggests urgent need for management, conservation, and research of these crayfishes. The presence of invasive crayfishes in particular seems strongly related to declines or local extirpations of *P. connectens* and *P. gambelii.* Accordingly, efforts to prevent the further introduction and spread of invasive crayfishes like *F. virilis,* or the “native invader” *P. leniusculus*, should be immediately implemented, and may include educational outreach or regulatory change and enforcement to prohibit these organisms from the live animal trade (Larson & Olden, 2011; Lodge et al., 2016). In areas where *F. virilis* or *P. leniusculus* are already present, management and maintenance of existing dispersal barriers such as dams and waterfalls may keep these invaders from spreading further and help to conserve existing pilose crayfish populations (Kerby et al., 2005; Fausch et al., 2009). In addition, where local conditions allow (e.g., small groundwater springs; Fig. 8), construction and maintenance of new dispersal barriers might be considered to protect extant *P. connectens* and *P. gambelii* populations (Cowart et al., 2018). Range declines of the pilose crayfishes were also seemingly associated with degraded stream benthic community condition (Hill et al., 2016). Management and regulation of point and nonpoint sources of water pollution or sedimentation may help to prevent current pilose crayfish habitat from also becoming highly degraded (Allan, 2004; Novotny & Smith, 2004; Strayer, 2006). Our SDM suggests that the pilose crayfishes most typically occur in the types of larger, low elevation, valley bottom streams that are at most risk of degradation from land use in our study region (Larson & Olden, 2011; Larson & Williams, 2015), and as such, persistence of these crayfishes is likely dependent on good management practices for water quality and in-stream habitat (Bilotta & Brazier, 2008; Strayer & Dudgeon, 2010).

We conclude by emphasizing that our study is the first dedicated to the ecology and distribution of the pilose crayfishes, but further basic distributional and ecological information is urgently needed to support the conservation of these species. We are relatively confident that we have sampled within the true historical range for both crayfishes, but aberrant occurrence records for each species across the larger western US merits investigation (Larson & Williams, 2015). *Pacifastacus connectens* and *P. gambelii* would certainly benefit from additional biological and ecological information, including life history studies (Moore, DiStefano & Larson, 2013), investigations of ecological interactions with other organisms, particularly invasive crayfishes (Usio, Konishi & Nakano, 2001; Pintor, Sih & Bauer, 2008), and habitat selection and use at finer grains than we could consider here (Flinders & Magoulick, 2007). We hope that our study will provide a baseline and motivation for future inquiry and conservation intervention for these interesting but minimally studied crayfishes.

##  Supplemental Information

10.7717/peerj.5668/supp-1 Table S1 Historical occurrences of the pilose crayfishes Table of historical *Pacifastacus connectens* and *Pacifastacus gambelii* occurrence records (*N* = 63) from various sources. Site number corresponds to our sampling sites in Table S2; those without site numbers were not sampled. Year collected is given when known. Additional information regarding source is given in footnotes.

10.7717/peerj.5668/supp-2 Table S2 Results of crayfish field sampling Table of field sampling results in the western US in the summers of 2016 and 2017, in order of date sampled. We provide latitudes and longitudes in North American Datum 1983 (NAD 83), whether the site was sampled using baited trapping or timed search, whether the site was lentic or lotic, crayfish species detected, and whether the site had a historical species record. Crayfish species detected include *Pacifastacus connectens* (*N* = 10), *Pacifastacus gambelii* (*N* = 10)*, Faxonius virilis* (*N* = 22), ** and *Pacifastacus leniusculus* (*N* = 29)*. *

10.7717/peerj.5668/supp-3 Figure S1 Sensitivity test of species distribution models Suitable crayfish habitat in the western United States predicted for combinations of background points (50, 300, and 1000) and pilose crayfish occurrence records (*P. connectens* individually*, P. gambelii* individually, and both pilose crayfish species combined). These predictions were developed from boosted regression tree models using historical crayfish occurrence records and the EarthEnv habitat layers (Domisch, Amatulli & Jetz, 2015). Our chosen model for main text analyses (1000 background points, both pilose crayfish species combined) is outlined in bold.

10.7717/peerj.5668/supp-4 Figure S2 Classification success of species distribution models Histograms showing number of true positives, true negatives, false positives, and false negatives for models with varying number of background points included (50, 300, and 1000) and pilose crayfish species included (*Pacifastacus connectens, Pacifastacus gambelii*, and pilose crayfish species modeled together). We chose to combine the individual models for *P. connectens* and *P. gambelii* here to simplify or standardize comparison to the single model of both pilose crayfishes together; if either individual *P. connectens* or *P. gambelii* predicted a location as suitable for one crayfish we accepted it as suitable for either, with absence locations where neither model predicted habitat as suitable for these crayfishes.

10.7717/peerj.5668/supp-5 Figure S3 Comparison of sampling method detections Frequency (number of sites) of crayfish catch-per-unit efforts (CPUE) for both timed searching and baited trapping across crayfish species sampled in this study. Crayfish were rarely detected with very low CPUE by timed searching, whereas crayfish were routinely detected with low CPUE by baited trapping. As such, we infer that poor detection probabilities for baited trapping of these crayfishes may have contributed to some observed false positives in comparison to our SDM (Fig. 4).

## Additional Information and Declarations

### Field Study Permissions

The following information was supplied relating to field study approvals (i.e., approving body and any reference numbers): Field sampling was conducted under Wyoming Game and Fish scientific collecting permit 33-1070, Idaho Department of Fish and Game permits F-16-32-16 and F-16-32-17, Utah Division of Wildlife Resources permit 2COLL9870, Nevada Department of Wildlife permit 428773, and Oregon Department of Fish and Wildlife permit 21325.

### Data Availability

The following information was supplied regarding data availability: The raw data collected by field sampling are provided in Table S2
