# Supplementary material for: Distribution, habitat associations, and conservation status updates for the pilose crayfish Pacifastacus gambelii (Girard, 1852) and Snake River pilose crayfish Pacifastacus connectens (Faxon, 1914) of the western United States
Source: PeerJ. 2018 Sep 27;6:e5668. doi: 10.7717/peerj.5668 (PMC6166635; doi:10.7717/peerj.5668)
Supplement: Table S1 — Table of historical Pacifastacus connectens and Pacifastacus gambelii occurrence records (N = 63) from various sources. Site number corresponds to our sampling sites in Table S2; those without site numbers were not sampled. Year collected is given when known. Additional information regarding source is given in footnotes. [file peerj-06-5668-s001.docx]

| **Site Number** | **Site Name** | **Latitude** | **Longitude** | **Historic Species Record** | **State** | **County** | **Source** | **Year Collected** |
| --- | --- | --- | --- | --- | --- | --- | --- | --- |
| 2 | Sulfur Creek Reservoir | 41.1471 | -110.8235 | *P. gambelii* | Wyoming | Uinta | Hubert (1988) | 1988 |
| 3 | Bear River State Park | 41.2606 | -110.9375 | *P. gambelii* | Wyoming | Uinta | Hubert (2010) | 1988 |
| 5 | Woodruff Narrows Reservoir | 41.5042 | -111.0211 | *P. gambelii* | Wyoming | Uinta | Hubert (1988) | 1988 |
| 8 | Bear Lake South | 41.8464 | -111.3381 | *P. gambelii* | Utah | Rich | Johnson (1986) | 1986 |
| 11 | Bear Lake North | 42.1198 | -111.2982 | *P. connectens* | Idaho | Bear Lake | Smithsonian Institution NMNH | 2001 |
| 17 | Salt Fork | 42.3993 | -111.0150 | *P. gambelii* | Wyoming | Lincoln | Pete Cavalli | 2012 |
| 25 | Polecat Creek | 44.1104 | -110.6884 | *P. gambelii* | Wyoming | Teton | Miller (1960) | <1960 |
| 34 | Snake River Roberts | 43.7216 | -112.0857 | *P. gambelii* | Idaho | Jefferson | Smithsonian Institution NMNH | 1949 |
| 37 | Falls Creek | 43.4416 | -111.3785 | *P. gambelii* | Idaho | Bonneville | Thomas Woolf | 2014 |
| 41 | Bear River below Alexander | 42.5936 | -111.7197 | *P. gambelii* | Idaho | Caribou | Smithsonian Institution NMNH | 1976 |
| 42 | Bear River Thatcher | 42.4082 | -111.7330 | *P. gambelii* | Idaho | Franklin | College of Idaho Museum | 1975 |
| 43 | Bear River Oneida | 42.2641 | -111.7527 | *P. gambelii* | Idaho | Franklin | Smithsonian Institution NMNH | 1975 |
| 44 | Bear River Preston | 42.0971 | -111.9165 | *P. gambelii* | Idaho | Franklin | Smithsonian Institution NMNH | 1976 |
| 52 | Sublett Creek | 42.3242 | -113.0482 | *P. gambelii* | Idaho | Cassia | Smithsonian Institution NMNH | 1981 |
| 53 | Massacre Rock | 42.6842 | -112.9814 | *P. gambelii* | Idaho | Power | Smithsonian Institution NMNH | 1950 |
| 56 | Cassia Creek | 42.2761 | -113.5152 | *P. gambelii* | Idaho | Cassia | Smithsonian Institution NMNH | 1980 |
| 57 | Raft River | 41.9522 | -113.6836 | *P. gambelii* | Utah | Box Elder | Johnson (1986) | 1984 |
| 58 | Rock Spring Creek | 41.7234 | -114.3766 | *P. gambelii* | Nevada | Elko | Jeff Petersen | 2014 |
| 63 | Trapper Creek | 42.1554 | -113.9985 | *P. gambelii* | Idaho | Cassia | College of Idaho Museum | 1990 |
| 66 | Rock Creek Lower | 42.5665 | -114.5030 | *P. gambelii* | Idaho | Twin Falls | College of Idaho Museum | 1987 |
| 68 | Vineyard Lake | 42.5905 | -114.3443 | *P. gambelii* | Idaho | Jerome | College of Idaho Museum | 1990 |
| 72 | Snake River Burley | 42.5560 | -113.7937 | *P. gambelii* | Idaho | Cassia | Smithsonian Institution NMNH | 1982 |
| 76 | Hyrum Reservoir | 41.6214 | -111.8539 | *P. gambelii* | Utah | Cache | Johnson (1986) | 1982 |
| 77 | Wellsville Reservoir | 41.6297 | -111.9294 | *P. gambelii* | Utah | Cache | Johnson (1986) | 1982 |
| 78 | Box Elder Creek | 41.4991 | -111.9835 | *P. gambelii* | Utah | Box Elder | Holt (1960) | 1958 |
| 79 | Weber River | 41.1145 | -111.7674 | *P. gambelii* | Utah | Morgan | Johnson (1986) | 1982 |
| 80 | Lost Creek Reservoir | 41.1867 | -111.3826 | *P. gambelii* | Utah | Morgan or Rich | Johnson (1986) | 1984 |
| 83 | Kimball | 40.7284 | -111.5366 | *P. gambelii* | Utah | Summit | Smithsonian Institution NMNH | 1976 |
| 102 | Snake River at Sidewinder Rapids | 42.8659 | -114.9069 | *P. connectens* | Idaho | Gooding | College of Idaho Museum | 2005 |
| 103 | Malad River Gorge | 42.8629 | -114.9029 | *P. connectens* | Idaho | Gooding | Bronwyn Williams | 2012 |
| 104 | Billingsley Creek | 42.8345 | -114.8904 | *P. connectens* | Idaho | Gooding | Smithsonian Institution NMNH | 1975 |
| 105 | Box Canyon Springs | 42.7075 | -114.8103 | *P. connectens* | Idaho | Gooding | College of Idaho Museum | 2007 |
| 106 | Sand Spring Creek | 42.7273 | -114.8352 | *P. connectens* | Idaho | Gooding | College of Idaho Museum | 1988 |
| 107 | Salmon Falls Creek | 42.6958 | -114.8558 | *P. connectens* | Idaho | Twin Falls | Smithsonian Institution NMNH | 1981 |
| 110 | Upper Salmon Falls Reservoir | 42.2092 | -114.7307 | *P. connectens* | Idaho | Twin Falls | Carnegie MNH | 1914 |
| 125 | Bruneau River near Bruneau | 42.8804 | -115.8180 | *P. connectens* | Idaho | Owyhee | Smithsonian Institution NMNH | 1969 |
| 136 | Crooked Creek | 42.8048 | -117.7350 | *P. connectens* | Oregon | Malheur | Miller (1960) | <1960 |
| 141 | Boulder Creek | 42.8244 | -116.7806 | *P. connectens* | Idaho | Owyhee | College of Idaho Museum | 1976 |
| 153 | Middle Fork Malheur River | 43.7354 | -118.3030 | *P. connectens* | Oregon | Harney | Miller (1960) | <1960 |
| 155 | Malheur River East of Juntura | 43.7823 | -118.0225 | *P. connectens* | Oregon | Harney | Smithsonian Institution NMNH | 1975 |
| 156 | Malheur River near Harper | 43.8577 | -117.6087 | *P. connectens* | Oregon | Harney | Smithsonian Institution NMNH | 1975 |
| 163 | Silver Creek | 43.2799 | -119.2483 | *P. connectens* | Oregon | Harney | Miller (1960) | <1960 |
| 164 | Golden Canal at Barnyard Springs | 43.2762 | -119.3102 | *P. connectens* | Oregon | Harney | Malheur NWF | 2012 |
| 165 | Double O Springs | 43.2804 | -119.3197 | *P. connectens* | Oregon | Harney | Malheur NWF | 2011 |
| 168 | Snake River near Nyssa | 43.8761 | -116.9846 | *P. connectens* | Oregon | Malheur | Miller (1960) | 1960 |
| 179 | Snake River Marsing | 43.5480 | -116.8012 | *P. connectens* | Idaho | Canyon | Smithsonian Institution NMNH | 1929 |
| 192 | Donner Und Blitzen River | 42.8007 | -118.8680 | *P. connectens* | Oregon | Harney | Miller (1960) | <1960 |
| 195 | Big Jacks Creek | 42.5940 | -115.9927 | *P. connectens* | Idaho | Owyhee | College of Idaho Museum | 1990 |
| 205 | Silvies River | 43.5989 | -119.0482 | *P. connectens* | Oregon | Harney | Miller (1960) | 1960 |
| 206 | South Fork Malheur River | 43.4080 | -118.2831 | *P. connectens* | Oregon | Harney | Smithsonian Institution NMNH | 1981 |
|  | Bear River | 42.2490 | -111.2717 | *P. gambelii* | Utah | Rich | Smithsonian Institution NMNH | 1975 |
|  | Big Creek | 41.6639 | -111.1990 | *P. gambelii* | Utah | Rich | Smithsonian Institution NMNH | 1976; 1983 |
|  | Crawfish Creek | 44.1515 | -110.6733 | *P. gambelii* | Wyoming | Teton | Miller (1960),  Smithsonian Institution NMNH | 1949, 1975 |
|  | Giraffe Creek | 42.4333 | -111.0095 | *P. gambelii* | Wyoming | Lincoln | Hubert (2010) | 2010 |
|  | Goose Creek | 41.9469 | -114.0758 | *P. gambelii* | Nevada | Elko | Miller (1960) | <1960 |
|  | Goose Creek | 42.0743 | -113.9312 | *P. gambelii* | Idaho | Cassia | Smithsonian Institution NMNH | 1980 |
|  | Marsh Creek | 42.4574 | -113.5194 | *P. gambelii* | Idaho | Cassia | Smithsonian Institution NMNH | 1950 |
|  | Rock Creek | 42.4544 | -114.3640 | *P. connectens* | Idaho | Twin Falls | College of Idaho Museum | 1985 |
|  | Salmon Falls Creek | 42.7115 | -114.8527 | *P. gambelii* | Idaho | Twin falls | Smithsonian Institution NMNH | 1976 |
|  | Snake River | 44.1416 | -110.6634 | *P. gambelii* | Wyoming | Teton | Miller (1960) | <1960 |
|  | Spring | 43.6232 | -110.6065 | *P. gambelii* | Wyoming | Teton | Hubert (2010) | 2010 |
|  | Wennergren's Pond | 41.7483 | -111.8683 | *P. gambelii* | Utah | Cache | Miller (1960) | <1960 |
|  | Stinking Lake Spring | 43.3266 | -119.3663 | *P. connectens* | Oregon | Harney | Smithsonian Institution NMNH | 1973 |

| Bronwyn Williams, North Carolina Museum of Natural Sciences  Carnegie Museum of Natural History  College of Idaho Orna J. Smith Museum of Natural History  Holt, P.C., 1960. The genus *Ceratodrilus* Hall, (Branchiobdellidae, Obligochaeta) with the description of a new species. Va. J. Sci. 11, 53–77.  Hubert, W.A., 2010. Survey of Wyoming crayfishes: 2007-2009. Cheyenne, WY.  Hubert, W.A., 1988. Survey of Wyoming crayfishes. Gt. Basin Nat. 48, 370–372.  Jeff Petersen, Nevada Department of Wildlife  Johnson, J.E., 1986. Inventory of Utah crayfish with notes on current distribution. Gt. Basin Nat. 46, 625–631.  Miller, G.C., 1960. The taxonomy and certain biological aspects of the crayfish of Oregon and Washington.  Malheur National Wildlife Refuge  Pete Cavalli, Wyoming Game and Fish Department  Smithsonian Institution National Museum of Natural History  Thomas Woolf, Idaho Department of Environmental Quality |
| --- |
