# Supplementary material for: Distribution, habitat associations, and conservation status updates for the pilose crayfish Pacifastacus gambelii (Girard, 1852) and Snake River pilose crayfish Pacifastacus connectens (Faxon, 1914) of the western United States
Source: PeerJ. 2018 Sep 27;6:e5668. doi: 10.7717/peerj.5668 (PMC6166635; doi:10.7717/peerj.5668)
Supplement: Table S2 — Table of field sampling results in the western US in the summers of 2016 and 2017, in order of date sampled. We provide latitudes and longitudes in North American Datum 1983 (NAD 83), whether the site was sampled using baited trapping or timed search, whether the site was lentic or lotic, crayfish species detected, and whether the site had a historical species record. Crayfish species detected include Pacifastacus connectens (N = 10), Pacifastacus gambelii (N = 10), Faxonius virilis (N = 22), and Pacifastacus leniusculus (N = 29). [file peerj-06-5668-s002.docx]

| **Site Number** | **Site Name** | **Date Sampled** | **Latitude** | **Longitude** | **Trapped/Timed** | **Lentic/Lotic** | **Crayfish Species Present** | **Historic Species Record** |
| --- | --- | --- | --- | --- | --- | --- | --- | --- |
| 1 | Bear River Upper | 7/16/2016 | 40.8731 | -110.8351 | Timed | Lotic |  |  |
| 2 | Sulfur Creek Reservoir | 7/16/2016 | 41.1471 | -110.8235 | Timed | Lentic | *F. virilis* | *P. gambelii* |
| 3 | Bear River State Park | 7/16/2016 | 41.2606 | -110.9375 | Timed | Lotic | *F. virilis* | *P. gambelii* |
| 4 | Bear River at Larson House | 7/16/2016 | 41.3929 | -111.0151 | Timed | Lotic | *F. virilis* |  |
| 5 | Woodruff Narrows Reservoir | 7/17/2016 | 41.5042 | -111.0211 | Timed | Lentic | *F. virilis* | *P. gambelii* |
| 6 | Woodruff Creek | 7/17/2016 | 41.4697 | -111.3032 | Timed | Lotic |  |  |
| 7 | Birch Creek | 7/17/2016 | 41.5072 | -111.3074 | Timed | Lotic |  |  |
| 86 | Woodruff Reservoir | 7/17/2016 | 41.4656 | -111.3205 | Timed | Lentic | *P. gambelii* |  |
| 8 | Bear Lake South | 7/18/2016 | 41.8464 | -111.3381 | Trapped | Lentic | *F. virilis* | *P. gambelii* |
| 9 | Beaver Creek | 7/18/2016 | 41.9616 | -111.5342 | Timed | Lotic |  |  |
| 10 | Fish Haven Creek | 7/18/2016 | 42.0400 | -111.4288 | Timed | Lotic |  |  |
| 11 | Bear Lake North | 7/19/2016 | 42.1198 | -111.2982 | Trapped | Lentic | *F. virilis* | *P. gambelii* |
| 12 | North Creek | 7/19/2016 | 42.3917 | -111.4928 | Trapped | Lotic |  |  |
| 14 | Montpelier Creek | 7/19/2016 | 42.3429 | -111.1765 | Timed | Lotic |  |  |
| 13 | Bear River Georgetown | 7/20/2016 | 42.4777 | -111.4058 | Trapped | Lotic | *F. virilis* |  |
| 15 | Smith's Fork | 7/20/2016 | 42.3775 | -110.8622 | Timed | Lotic |  |  |
| 16 | Raymond Creek | 7/20/2016 | 42.2768 | -111.0221 | Timed | Lotic |  |  |
| 17 | Salt Fork | 7/20/2016 | 42.3993 | -111.0150 | Timed | Lotic | *P. gambelii* | *P. gambelii* |
| 18 | Swift Creek | 7/21/2016 | 42.7258 | -110.9023 | Trapped | Lotic |  |  |
| 19 | Tincup Creek | 7/21/2016 | 42.9782 | -111.1683 | Timed | Lotic | *P. gambelii* |  |
| 20 | McCoy Creek | 7/21/2016 | 43.1805 | -111.1245 | Timed | Lotic |  |  |
| 25 | Polecat Creek | 7/22/2016 | 44.1104 | -110.6884 | Timed | Lotic |  | *P. gambelii* |
| 26 | Buffalo Fork | 7/22/2016 | 43.8581 | -110.2738 | Timed | Lotic |  |  |
| 27 | Lower Slide Lake | 7/22/2016 | 43.6371 | -110.5254 | Trapped | Lentic |  |  |
| 87 | Upper Snake River | 7/23/2016 | 43.3007 | -110.7751 | Timed | Lotic |  |  |
| 88 | Greys River | 7/23/2016 | 43.0735 | -110.8376 | Timed | Lotic |  |  |
| 28 | Teton River | 7/25/2016 | 43.7527 | -111.2052 | Trapped | Lotic |  |  |
| 29 | Conant Creek | 7/25/2016 | 44.0050 | -111.1492 | Timed | Lotic |  |  |
| 30 | Warm River | 7/25/2016 | 44.2050 | -111.2518 | Timed | Lotic |  |  |
| 31 | Big Spring | 7/26/2016 | 44.4996 | -111.2555 | Trapped | Lotic |  |  |
| 32 | Lower Henry's Fork | 7/26/2016 | 44.0697 | -111.5106 | Timed | Lotic |  |  |
| 33 | Lower Teton River | 7/26/2016 | 43.9341 | -111.6094 | Timed | Lotic |  |  |
| 34 | Snake River Roberts | 7/27/2016 | 43.7216 | -112.0857 | Trapped | Lotic | *F. virilis* | *P. gambelii* |
| 35 | Snake River Twin Bridges | 7/27/2016 | 43.6726 | -111.7685 | Trapped | Lotic |  |  |
| 37 | Falls Creek | 7/27/2016 | 43.4416 | -111.3785 | Timed | Lotic | *P. gambelii* | *P. gambelii* |
| 90 | Burns Creek | 7/27/2016 | 43.6052 | -111.4675 | Timed | Lotic |  |  |
| 36 | Ririe Reservoir | 7/28/2016 | 43.5761 | -111.7347 | Trapped | Lentic | *P. leniusculus* |  |
| 38 | Blackfoot River | 7/28/2016 | 43.0443 | -111.9110 | Timed | Lotic | *P. gambelii* |  |
| 39 | Pebble Creek | 7/28/2016 | 42.7343 | -112.0258 | Timed | Lotic |  |  |
| 40 | Portneuf River Lava | 7/29/2016 | 42.6432 | -112.0061 | Trapped | Lotic | *P. gambelii* |  |
| 41 | Bear River below Alexander | 7/29/2016 | 42.5936 | -111.7197 | Trapped | Lotic | *F. virilis* | *P. gambelii* |
| 43 | Bear River Oneida | 7/29/2016 | 42.2641 | -111.7527 | Timed | Lotic | *F. virilis* | *P. gambelii* |
| 42 | Bear River Thatcher | 7/30/2016 | 42.4082 | -111.7330 | Trapped | Lotic |  | *P. gambelii* |
| 44 | Bear River Preston | 7/30/2016 | 42.0971 | -111.9165 | Trapped | Lotic |  | *P. gambelii* |
| 45 | Cub River | 7/30/2016 | 42.1404 | -111.6286 | Trapped | Lotic |  |  |
| 46 | Deep Creek Reservoir | 7/30/2016 | 42.2110 | -112.1690 | Timed | Lentic |  |  |
| 48 | Portneuf River Crane | 7/30/2016 | 42.7243 | -112.2089 | Timed | Lotic | *P. gambelii* |  |
| 49 | Portneuf River Pocatello | 7/30/2016 | 42.8213 | -112.4055 | Timed | Lotic | *F. virilis* |  |
| 50 | Knox Creek | 8/1/2016 | 42.5062 | -112.6576 | Timed | Lotic |  |  |
| 51 | Stone Reservoir | 8/1/2016 | 42.0717 | -112.6919 | Timed | Lentic |  |  |
| 52 | Sublett Creek | 8/1/2016 | 42.3242 | -113.0482 | Timed | Lotic |  | *P. gambelii* |
| 53 | Massacre Rock | 8/2/2016 | 42.6842 | -112.9814 | Trapped | Lotic | *F. virilis* | *P. gambelii* |
| 54 | Register Rock | 8/2/2016 | 42.6529 | -113.0172 | Trapped | Lotic | *F. virilis* |  |
| 57 | Raft River | 8/2/2016 | 41.9522 | -113.6836 | Timed | Lotic | *P. gambelii* | *P. gambelii* |
| 56 | Cassia Creek | 8/3/2016 | 42.2761 | -113.5152 | Trapped | Lotic |  | *P. gambelii* |
| 58 | Rock Spring Creek | 8/3/2016 | 41.7234 | -114.3766 | Timed | Lotic | *P. gambelii* | *P. gambelii* |
| 62 | Goose Creek Reservoir | 8/4/2016 | 42.1841 | -113.9311 | Timed | Lentic | *F. virilis* |  |
| 63 | Trapper Creek | 8/4/2016 | 42.1554 | -113.9985 | Timed | Lotic |  | *P. gambelii* |
| 92 | Cassia Creek 2 | 8/4/2016 | 42.2410 | -113.5718 | Trapped | Lotic |  |  |
| 64 | Rock Creek Upper | 8/5/2016 | 42.3249 | -114.2731 | Timed | Lotic |  |  |
| 66 | Rock Creek Lower | 8/5/2016 | 42.5665 | -114.5030 | Timed | Lotic |  | *P. gambelii* |
| 69 | Dierkes Lake | 8/5/2016 | 42.5947 | -114.3915 | Trapped | Lentic | *F. virilis* |  |
| 70 | Shoshone Falls | 8/5/2016 | 42.5963 | -114.3981 | Trapped | Lotic |  |  |
| 71 | Snake River Murtaugh | 8/6/2016 | 42.4991 | -114.1520 | Timed | Lotic | *F. virilis, P. gambelii* |  |
| 68 | Vineyard Lake | 8/6/2016 | 42.5905 | -114.3443 | Trapped | Lentic |  | *P. gambelii* |
| 72 | Snake River Burley | 8/6/2016 | 42.5560 | -113.7937 | Trapped | Lotic | *F. virilis* | *P. gambelii* |
| 74 | Logan River | 8/7/2016 | 41.7437 | -111.7593 | Timed | Lotic |  |  |
| 75 | Little Bear River | 8/7/2016 | 41.5178 | -111.7667 | Timed | Lotic |  |  |
| 73 | Cutler Reservoir | 8/7/2016 | 41.7863 | -111.9560 | Trapped | Lentic |  |  |
| 94 | Bear River Cache | 8/7/2016 | 41.8013 | -111.9096 | Trapped | Lotic |  |  |
| 78 | Box Elder Creek | 8/8/2016 | 41.4991 | -111.9835 | Timed | Lotic |  | *P. gambelii* |
| 79 | Weber River | 8/8/2016 | 41.1145 | -111.7674 | Timed | Lotic |  | *P. gambelii* |
| 80 | Lost Creek Reservoir | 8/8/2016 | 41.1867 | -111.3826 | Timed | Lentic | *F. virilis* | *P. gambelii* |
| 76 | Hyrum Reservoir | 8/8/2016 | 41.6214 | -111.8539 | Trapped | Lentic |  | *P. gambelii* |
| 77 | Wellsville Reservoir | 8/8/2016 | 41.6297 | -111.9294 | Trapped | Lentic |  | *P. gambelii* |
| 82 | Weber River Coalville | 8/10/2016 | 40.9117 | -111.4062 | Timed | Lotic |  |  |
| 83 | Kimball | 8/10/2016 | 40.7284 | -111.5366 | Timed | Lotic |  | *P. gambelii* |
| 85 | Beaver Creek Kamas | 8/10/2016 | 40.6308 | -111.1868 | Timed | Lotic |  |  |
| 103 | Malad River Gorge | 7/2/2017 | 42.8629 | -114.9029 | Timed | Lotic | *P. connectens* | *P. connectens* |
| 101 | Little Wood River | 7/3/2017 | 43.0422 | -114.1538 | Timed | Lotic | *F. virilis* |  |
| 102 | Snake River at Sidewinder Rapids | 7/3/2017 | 42.8659 | -114.9069 | Trapped | Lotic |  | *P. connectens* |
| 104 | Billingsley Creek | 7/3/2017 | 42.8345 | -114.8904 | Trapped | Lotic |  | *P. connectens* |
| 106 | Sand Spring Creek | 7/3/2017 | 42.7273 | -114.8352 | Timed | Lotic | *P. connectens* | *P. connectens* |
| 108 | Niagara Spring | 7/3/2017 | 42.6630 | -114.6743 | Timed | Lotic | *P. connectens* |  |
| 105 | Box Canyon Springs | 7/4/2017 | 42.7075 | -114.8103 | Timed | Lotic | *P. connectens* | *P. connectens* |
| 107 | Salmon Falls Creek | 7/4/2017 | 42.6958 | -114.8558 | Timed | Lotic |  | *P. connectens* |
| 184 | Snake River Thousand Springs | 7/4/2017 | 42.7426 | -114.8484 | Trapped | Lotic |  |  |
| 109 | Salmon Falls Creek | 7/5/2017 | 42.5422 | -114.9498 | Timed | Lotic |  |  |
| 110 | Upper Salmon Falls Reservoir | 7/5/2017 | 42.2092 | -114.7307 | Timed | Lentic | *F. virilis* | *P. connectens* |
| 111 | Cedar Creek Reservoir | 7/5/2017 | 42.2020 | -114.8983 | Timed | Lentic | *F. virilis* |  |
| 112 | Snake River near Glenns Ferry | 7/6/2017 | 42.9341 | -115.3219 | Timed | Lotic |  |  |
| 113 | Bruneau River | 7/7/2017 | 42.6517 | -115.7015 | Timed | Lotic |  |  |
| 115 | Clover Creek | 7/8/2017 | 42.4434 | -115.3694 | Timed | Lotic |  |  |
| 116 | East Fork Jarbidge River | 7/8/2017 | 42.0311 | -115.3676 | Timed | Lotic |  |  |
| 114 | Bruneau Dunes Lake | 7/8/2017 | 42.8954 | -115.6964 | Trapped | Lentic |  |  |
| 125 | Bruneau River near Bruneau | 7/8/2017 | 42.8804 | -115.8180 | Trapped | Lotic | *P. leniusculus* | *P. connectens* |
| 117 | Meadow Creek | 7/9/2017 | 41.9063 | -115.6781 | Timed | Lotic |  |  |
| 119 | Owyhee River near Wild Horse | 7/9/2017 | 41.7261 | -115.8954 | Timed | Lotic | *P. leniusculus* |  |
| 120 | Wilson Reservoir | 7/10/2017 | 41.6706 | -116.3376 | Timed | Lentic |  |  |
| 122 | Sheep Creek | 7/10/2017 | 42.2050 | -115.7435 | Timed | Lotic |  |  |
| 118 | Wild Horse Reservoir | 7/10/2017 | 41.6670 | -115.8017 | Trapped | Lentic |  |  |
| 124 | West Shoofly Creek | 7/11/2017 | 42.7484 | -116.2304 | Timed | Lotic |  |  |
| 126 | Rattlesnake Creek | 7/12/2017 | 43.2113 | -115.5611 | Timed | Lotic | *P. leniusculus* |  |
| 127 | Little Camas Reservoir | 7/12/2017 | 43.3479 | -115.3904 | Timed | Lotic |  |  |
| 128 | Anderson Ranch Reservoir | 7/12/2017 | 43.3992 | -115.4136 | Timed | Lentic | *P. leniusculus* |  |
| 185 | South Fork Boise River | 7/13/2017 | 43.6040 | -115.0797 | Trapped | Lotic |  |  |
| 186 | Soldier Creek | 7/13/2017 | 43.4870 | -114.8284 | Timed | Lotic |  |  |
| 187 | Moonstone, Magic Reservoir | 7/13/2017 | 43.3353 | -114.4328 | Timed | Lentic |  |  |
| 131 | Indian Creek Reservoir | 7/14/2017 | 43.3863 | -116.0139 | Timed | Lentic | *F. virilis* |  |
| 132 | Mores Creek | 7/14/2017 | 43.6644 | -115.9787 | Timed | Lotic | *P. leniusculus* |  |
| 196 | Camas Creek | 7/14/2017 | 43.2877 | -114.8020 | Trapped | Lotic |  |  |
| 188 | Lucky Peak | 7/15/2017 | 43.5253 | -116.0641 | Trapped | Lotic |  |  |
| 134 | Jordan Creek | 7/16/2017 | 43.0153 | -116.7307 | Timed | Lotic |  |  |
| 141 | Boulder Creek | 7/16/2017 | 42.8244 | -116.7806 | Timed | Lotic |  | *P. connectens* |
| 138 | Three Forks | 7/17/2017 | 42.5473 | -117.1707 | Timed | Lotic |  |  |
| 142 | Cow Lake | 7/17/2017 | 43.0956 | -117.3310 | Timed | Lentic |  |  |
| 135 | Antelope Reservoir | 7/17/2017 | 42.9084 | -117.2366 | Trapped | Lentic |  |  |
| 136 | Crooked Creek | 7/17/2017 | 42.8048 | -117.7350 | Trapped | Lotic | *P. connectens* | *P. connectens* |
| 189 | Owyhee River near Rome | 7/17/2017 | 42.8363 | -117.6219 | Trapped | Lotic |  |  |
| 139 | Current Creek | 7/18/2017 | 42.5705 | -116.7201 | Timed | Lotic |  |  |
| 180 | Jump Creek | 7/18/2017 | 43.4799 | -116.9227 | Timed | Lotic | *P. leniusculus* |  |
| 190 | North Fork Owyhee River | 7/18/2017 | 42.5918 | -116.9813 | Trapped | Lotic |  |  |
| 201 | Bully Creek | 7/22/2017 | 44.0257 | -117.4556 | Timed | Lotic | *P. leniusculus* |  |
| 202 | Owyhee River at Snively Hot Springs | 7/23/2017 | 43.7308 | -117.2037 | Timed | Lotic | *P. leniusculus* |  |
| 203 | North Fork Malheur River | 7/24/2017 | 43.9727 | -118.1941 | Timed | Lotic | *P. leniusculus* |  |
| 176 | Boise River South | 7/25/2017 | 43.6396 | -116.2447 | Trapped | Lotic |  |  |
| 177 | Boise River North | 7/25/2017 | 43.6619 | -116.2818 | Trapped | Lotic |  |  |
| 144 | Succor Creek | 7/25/2017 | 43.4539 | -117.1195 | Timed | Lotic | *P. leniusculus* |  |
| 145 | Owyhee Lake/Reservoir | 7/25/2017 | 43.6232 | -117.2376 | Timed | Lentic | *P. leniusculus* |  |
| 197 | Cottonwood Creek | 7/25/2017 | 43.7401 | -117.6739 | Timed | Lotic | *P. connectens* |  |
| 152 | Warm Springs Reservoir | 7/26/2017 | 43.6032 | -118.2418 | Timed | Lentic | *P. leniusculus* |  |
| 153 | Middle Fork Malheur River | 7/26/2017 | 43.7354 | -118.3030 | Timed | Lotic | *P. leniusculus* | *P. connectens* |
| 204 | Pine Creek | 7/26/2017 | 43.7823 | -118.5791 | Timed | Lotic | *P. leniusculus* |  |
| 205 | Silvies River | 7/26/2017 | 43.5989 | -119.0482 | Timed | Lotic | *P. leniusculus* | *P. connectens* |
| 206 | South Fork Malheur River | 7/26/2017 | 43.4080 | -118.2831 | Timed | Lotic | *P. leniusculus* | *P. connectens* |
| 159 | Sawtooth Creek | 7/27/2017 | 43.8421 | -119.3086 | Timed | Lotic |  |  |
| 154 | Beulah Reservoir | 7/27/2017 | 43.9114 | -118.1526 | Trapped | Lentic |  |  |
| 155 | Malheur River East of Juntura | 7/27/2017 | 43.7823 | -118.0225 | Trapped | Lotic | *P. leniusculus* | *P. connectens* |
| 191 | Upper Silvies River | 7/27/2017 | 44.1968 | -119.1808 | Timed | Lotic | *P. leniusculus* |  |
| 207 | Silver Creek | 7/27/2017 | 43.7015 | -119.6373 | Timed | Lotic | *P. leniusculus* |  |
| 208 | Emmigrant Creek | 7/27/2017 | 43.8680 | -119.4155 | Timed | Lotic | *P. leniusculus* |  |
| 165 | Double O Springs | 7/28/2017 | 43.2804 | -119.3197 | Timed | Lotic | *P. connectens* | *P. connectens* |
| 192 | Donner Und Blitzen River | 7/28/2017 | 42.8007 | -118.8680 | Timed | Lotic | *P. leniusculus* | *P. connectens* |
| 209 | Donner Und Blitzen River | 7/28/2017 | 42.6356 | -118.7644 | Timed | Lotic | *P. leniusculus* |  |
| 148 | East Canal, Page Springs | 7/29/2017 | 42.8093 | -118.8687 | Timed | Lotic |  |  |
| 146 | Krumbo Reservoir | 7/29/2017 | 42.9514 | -118.8060 | Trapped | Lentic |  |  |
| 163 | Silver Creek | 7/29/2017 | 43.2799 | -119.2483 | Trapped | Lotic | *P. connectens* | *P. connectens* |
| 164 | Golden Canal at Barnyard Springs | 7/29/2017 | 43.2762 | -119.3102 | Trapped | Lotic | *P. connectens* | *P. connectens* |
| 194 | Malheur Reservoir | 7/30/2017 | 44.3607 | -117.6828 | Timed | Lentic |  |  |
| 193 | Willow Creek | 7/30/2017 | 44.2859 | -117.5500 | Timed | Lotic | *P. leniusculus* |  |
| 210 | Summit Creek | 7/30/2017 | 44.1275 | -118.5786 | Timed | Lotic | *P. leniusculus* |  |
| 211 | Little Malheur River | 7/30/2017 | 44.2329 | -118.2487 | Timed | Lotic | *P. leniusculus* |  |
| 175 | Payette River near Crouch | 7/31/2017 | 44.1135 | -115.9706 | Timed | Lotic |  |  |
| 156 | Malheur River near Harper | 7/31/2017 | 43.8577 | -117.6087 | Trapped | Lotic |  | *P. connectens* |
| 168 | Snake River near Nyssa | 7/31/2017 | 43.8761 | -116.9846 | Trapped | Lotic |  | *P. connectens* |
| 169 | Snake River near Ontario | 7/31/2017 | 44.0491 | -116.9740 | Trapped | Lotic |  |  |
| 173 | Payette River | 8/1/2017 | 43.8962 | -116.6270 | Trapped | Lotic | *P. leniusculus* |  |
| 174 | Payette River near Montour | 8/1/2017 | 43.9310 | -116.3360 | Trapped | Lotic | *P. leniusculus* |  |
| 133 | Snake River Swan Falls | 8/2/2017 | 43.2460 | -116.3806 | Trapped | Lotic |  |  |
| 178 | Lowell Lake | 8/2/2017 | 43.5610 | -116.6602 | Trapped | Lentic |  |  |
| 179 | Snake River Marsing | 8/2/2017 | 43.5480 | -116.8012 | Trapped | Lotic |  | *P. connectens* |
| 167 | Boise River near Caldwell | 8/2/2017 | 43.6886 | -116.6861 | Trapped | Lotic | *P. leniusculus* |  |
| 195 | Big Jacks Creek | 8/3/2017 | 42.5940 | -115.9927 | Timed | Lotic | *P. connectens* | *P. connectens* |
