# Supplementary figures and images for: Distribution, habitat associations, and conservation status updates for the pilose crayfish Pacifastacus gambelii (Girard, 1852) and Snake River pilose crayfish Pacifastacus connectens (Faxon, 1914) of the western United States
Source: PeerJ. 2018 Sep 27;6:e5668. doi: 10.7717/peerj.5668 (PMC6166635; doi:10.7717/peerj.5668)

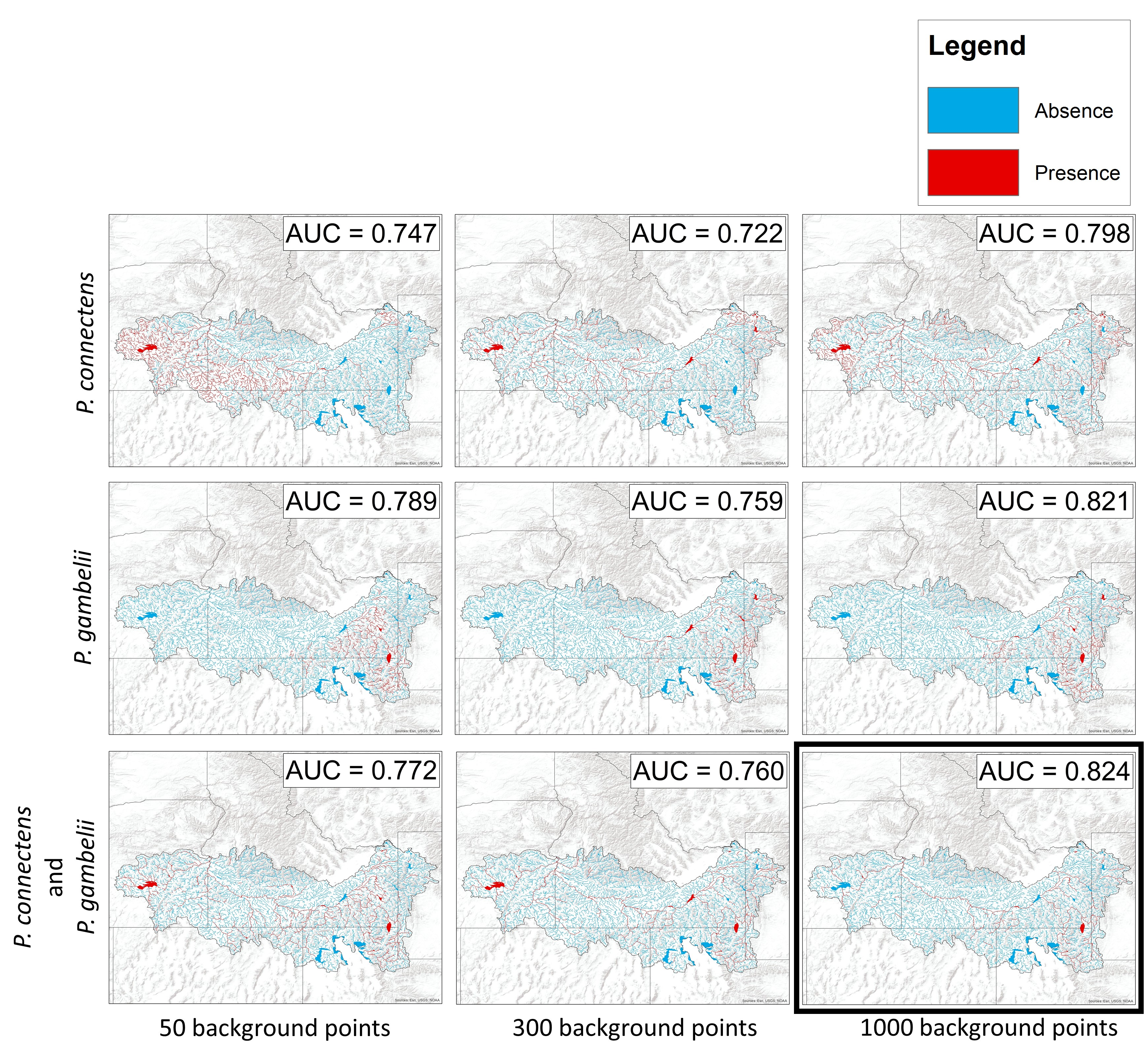

Supplement: Figure S1 — Suitable crayfish habitat in the western United States predicted for combinations of background points (50, 300, and 1000) and pilose crayfish occurrence records (P. connectens individually, P. gambelii individually, and both pilose crayfish species combined). These predictions were developed from boosted regression tree models using historical crayfish occurrence records and the EarthEnv habitat layers (Domisch, Amatulli & Jetz, 2015). Our chosen model for main text analyses (1000 background points, both pilose crayfish species combined) is outlined in bold. [file peerj-06-5668-s003.png]

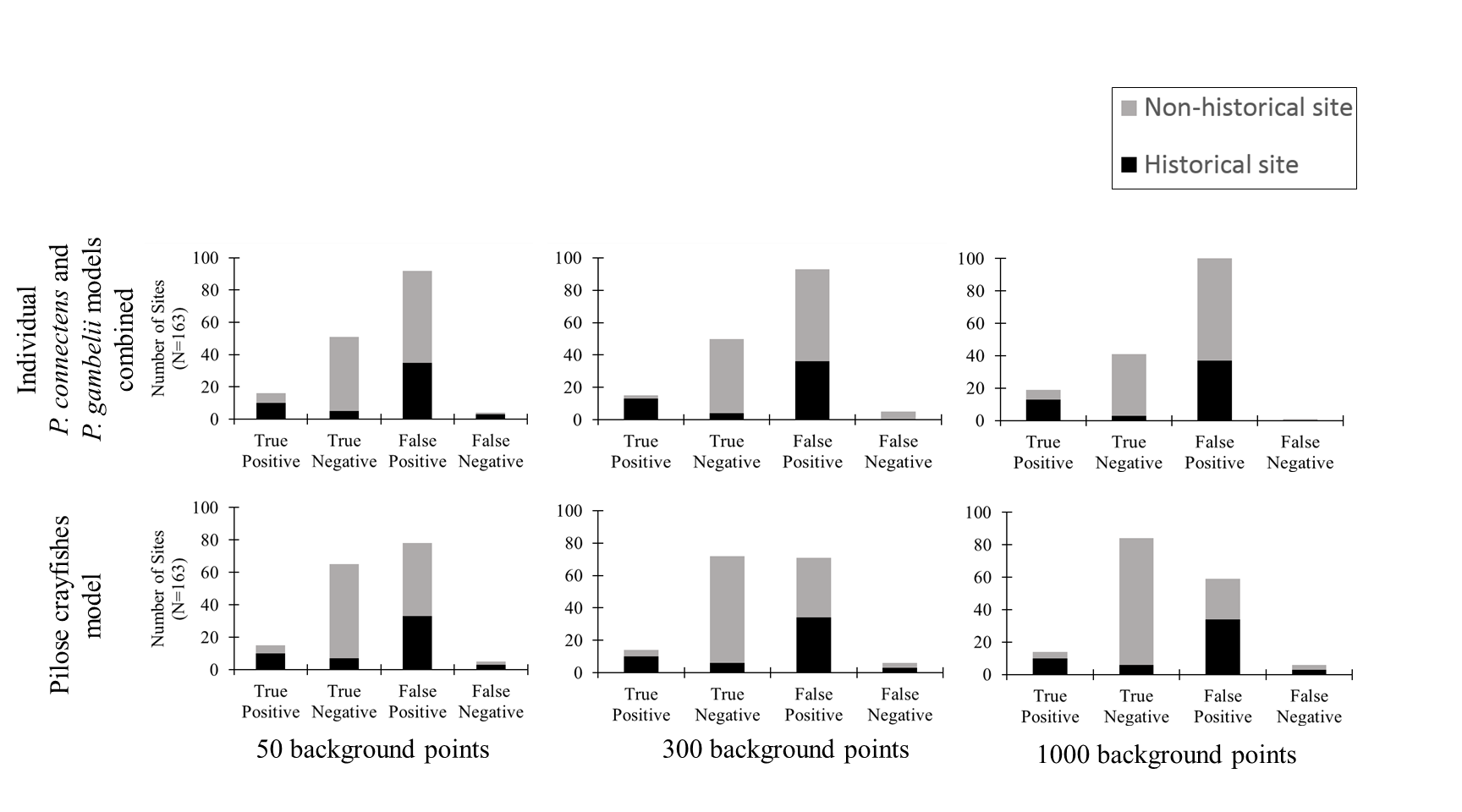

Supplement: Figure S2 — Histograms showing number of true positives, true negatives, false positives, and false negatives for models with varying number of background points included (50, 300, and 1000) and pilose crayfish species included (Pacifastacus connectens, Pacifastacus gambelii, and pilose crayfish species modeled together). We chose to combine the individual models for P. connectens and P. gambelii here to simplify or standardize comparison to the single model of both pilose crayfishes together; if either individual P. connectens or P. gambelii predicted a location as suitable for one crayfish we accepted it as suitable for either, with absence locations where neither model predicted habitat as suitable for these crayfishes. [file peerj-06-5668-s004.png]

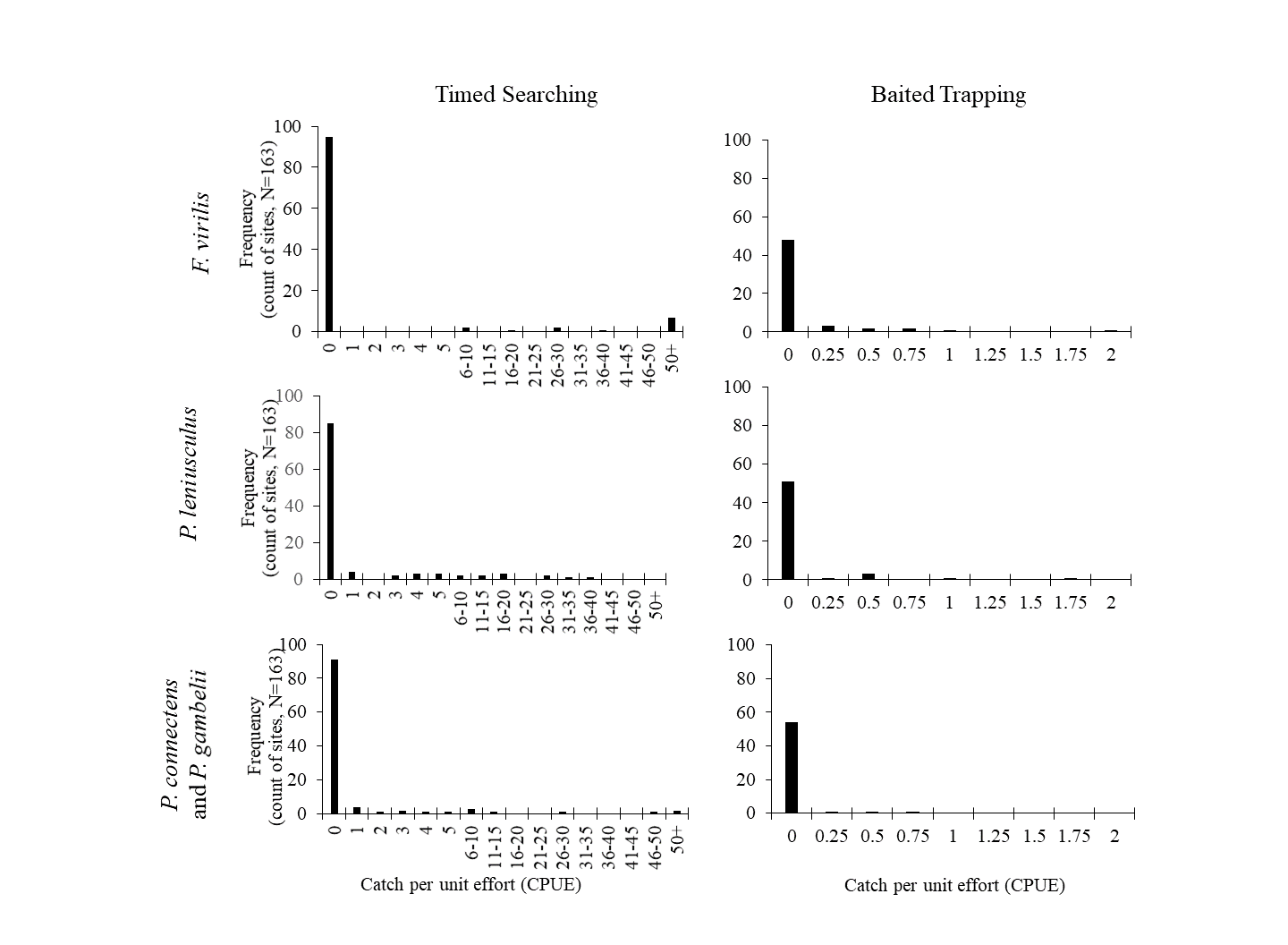

Supplement: Figure S3 — Frequency (number of sites) of crayfish catch-per-unit efforts (CPUE) for both timed searching and baited trapping across crayfish species sampled in this study. Crayfish were rarely detected with very low CPUE by timed searching, whereas crayfish were routinely detected with low CPUE by baited trapping. As such, we infer that poor detection probabilities for baited trapping of these crayfishes may have contributed to some observed false positives in comparison to our SDM (Fig. 4). [file peerj-06-5668-s005.png]
